# Evolutionary characteristics, expression patterns of wheat receptor-like kinases and functional analysis of *TaCrRLK1L16*

**DOI:** 10.1007/s44154-025-00215-y

**Published:** 2025-04-03

**Authors:** Guosen Zhao, Shiao Qin, Zhimin Wei, Xingxuan Bai, Jia Guo, Zhensheng Kang, Jun Guo

**Affiliations:** State Key Laboratory for Crop Stress Resistance and High-Efficiency Production, Key Laboratory of Plant Protection Resources and Pest Management of Ministry of Education, College of Plant Protection, Northwest A&F University, Yangling, Shaanxi, 712100 China

**Keywords:** Wheat, Receptor-like kinase, *Puccinia striiformis* f. sp. *tritici*, Plant immunity

## Abstract

Reverse genetics research in complex hexaploid wheat often encounters challenges in determining the priority of gene functional characterization. This study aims to systematically analyze the wheat (*Triticum aestivum*) receptor-like kinase (*TaRLK*) gene family and develop an effective strategy to identify key candidate genes for further investigation. We identified 3,424 *TaRLKs* using bioinformatics methods and analyzed the diverse and conserved evolutionary relationships of *RLKs* among Arabidopsis, rice and wheat. Based on publicly available and our laboratory’s transcriptome data, we comprehensively analyzed the transcriptional expression patterns of *TaRLKs* in response to various stresses, particularly *Puccinia striiformis* f. sp. *tritici* (*Pst*). The *TaCrRLK1L16*, which is upregulated during *Pst* infection and triggered cell death in *Nicotiana benthamiana*, has been identified as a key candidate gene for further functional characterization. Furthermore, our results suggested that the transgenic wheat overexpressing *TaCrRLK1L16* significantly enhanced resistance to *Pst*. This study will provide valuable insights into understanding the evolutionary characteristics and expression patterns of *TaRLKs* while offering a novel strategy for determining the priority of key candidate *TaRLKs*.

**Supplementary Information:**

The online version contains supplementary material available at 10.1007/s44154-025-00215-y.

## Introduction

During plant growth and development, they are frequently attacked by various pathogenic microorganisms including fungi, bacteria, viruses and oomycetes (Teixeira et al. 2019). These pathogens employ diverse mechanisms to penetrate the physical barrier of plant tissue, utilize various strategies to colonize host plants for acquiring essential nutrients necessary for their growth, and ultimately cause damage to host tissue by secreting pathogenic factors (Dou and Zhou 2012). Unlike mammals, plants lack a circulating immune system. However, they possess cell surface receptor-like kinases (RLKs), functionally analogous to animal receptor tyrosine kinases (RTKs), which play pivotal roles in initiating plant immune responses (Dangl and Jones 2001; Ausubel 2005; Chisholm et al. 2006). Typically, an RLK contains an N-terminal signal peptide sequence, various extracellular ligand-binding domains, a single transmembrane domain, and an intracellular C-terminal domain with eukaryotic protein kinase characteristics similar to RTKs (Schlessinger 2014). RLKs containing only the intracellular kinase domain were defined as receptor-like cytoplasmic kinases (RLCKs). Plants have evolved a two-layer immune system to recognize and defend against pathogens: pattern-triggered immunity (PTI) and effector-triggered immunity (ETI) (Zipfel 2014). Pattern recognition receptors (PRRs) are key components that recognize extracellular molecular patterns and activate intracellular signaling pathways. RLCKs act as regulators of multiple intracellular signal nodes through conserved phosphorylation cascades (Liang and Zhang 2022; Rao et al. 2018). PRRs and RLCKs are essential for balancing plant growth and development with immunity and orchestrating complex defense responses against microbial pathogens (Liang and Zhou 2018).

Wheat faces threats from various pathogens globally. Given that the pace of breeding for disease resistance in wheat lags behind the rate of pathogen evolution, there is an urgent need to integrate multi-omics data and adopt novel strategies to identify resistance gene with potential applications in the complex wheat genome (Zhao and Kang 2023). Recent evidence highlights the pivotal roles of wheat receptor-like kinases in responses to biotic stress. For instance, leucine-rich repeat receptor–like kinases, such as TaXa21 and TaSERK1, positively regulate high-temperature seedling plant resistance to *Pst* (Wang et al. 2019; Shi et al. 2023). Similarly, cysteine-rich receptor-like kinases, including TaCRK2 and TaRLK-R1/2/3, contribute to rust resistance in wheat (Zhou et al. 2007; Gu et al. 2020). A conserved wall-associated receptor kinase (WAK)-like protein, *Stb6,* controls gene-for-gene resistance to *Zymoseptoria tritici* (Saintenac et al. 2021). Notably, an atypical cysteine-rich receptor-like kinase fusion protein, WTK6-vWA (*Lr9*), confers leaf rust resistance in wheat. This fusion protein encodes a unique disease resistance protein with two tandem repeat kinases at the N-terminus and von Willebrand factor A (vWA) and Vwaint domains at the C-terminus (Wang et al. 2023). Over the past decades, *Catharanthus roseus* receptor-like kinase 1-like (CrRLK1L) protein and their rapid alkalinization factor (RALF) peptide ligands have attracted considerable attention (Zhang et al. 2023; Huang et al. 2024). Among these, FERONIA, a CrRLK1L protein, is central to multiple physiological processes mediated by RALF signaling, including cell growth, cell wall integrity surveillance, RNA and energy metabolism, plant hormone regulation and stress responses (Zhu et al. 2021). Extracellular pectin-RALF phase separation mediates FERONIA’s global signaling functions (Liu et al. 2024a, b). Overexpression of TaFER^ECD^ confers resistance to *Fusarium graminearum* in *Arabidopsis* without compromising growth (Wang et al. 2024). In addition to identifying resistance genes, modifying and editing plant susceptible genes offers new avenues to enhance crop resistance. For example, the receptor-like cytoplasmic kinase *TaPsIPK1* mediates susceptibility to *Pst*. CRISPR-Cas9 inactivation of *TaPsIPK1* in wheat confers robust broad-spectrum resistance without growth and yield penalty (Wang et al. 2022; Macho et al. 2022). These studies provide valuable genetic resources for accelerating plant disease resistance breeding, and also offer new insights into plant *RLKs*-mediated immune signal. Nevertheless, the functions of many *TaRLKs* in disease resistance remain uncharacterized, and the regulatory network underlying *TaRLKs*-mediated immunity require further elucidation.

Although advancements in wheat multi-omics data provide valuable support for the identification and analysis of key disease resistance genes, characterizing the functions of large gene families in wheat remains a significant challenge (Wu et al. 2023; Gan et al. 2024). Increasing evidence suggests that plant immune responses are often associated with a form of regulated cell death known as the hypersensitive response (HR) (Pitsili et al. 2020). For example, enhanced expression of *CRK28* in Arabidopsis increased resistance to *Pseudomonas syringae*, while its expression in *N. benthamiana* induced cell death (Yadeta et al. 2017). Similarly, the GmLecRKs-GmCDL1-MAPK module plays a crucial role in soybean resistance to *Phytophthora sojae* and nematodes; overexpression of *GmLecRK08g* in *N. benthamiana* resulted in pronounced cell death accompanied by high H_2_O_2_ accumulation (Zhang et al. 2024). Therefore, the candidate genes identified through multi-omics data analysis can be transiently overexpressed in *N. benthamiana*. Observing the resulting HR phenotype allows for the rapid identification of genes that may play key roles in plant disease resistance.

In this study, we identified 3,424 *TaRLKs* in wheat and conducted a comprehensive analysis of their physicochemical properties, phylogeny, orthologous groups, selection pressure, evolutionary characteristics and conserved kinase activity motifs. Additionally, we analyzed the expression patterns of *TaRLKs* across different tissues and under different biotic and abiotic stresses, with a particular focus on transcriptional level to *Pst* infection, utilized publicly available transcriptome data as well as previously published data from our laboratory. Our results suggested that *TaCrRLK1L16* exhibited the highest expression level induced by *Pst* in the CrRLK1L subfamily and it can trigger cell death in *N. benthamiana*. Consequently, we centered our investigation on *TaCrRLK1L16* and characterized its functional role in the wheat-*Pst* interaction. This study offers valuable insights into the evolutionary characteristics and functional roles of *TaRLKs*, contributing to a deeper understanding of their involvement in plant immune responses.

## Results

### Identification, classification, and evolutionary analysis of *TaRLKs*

**Fig. 1:**
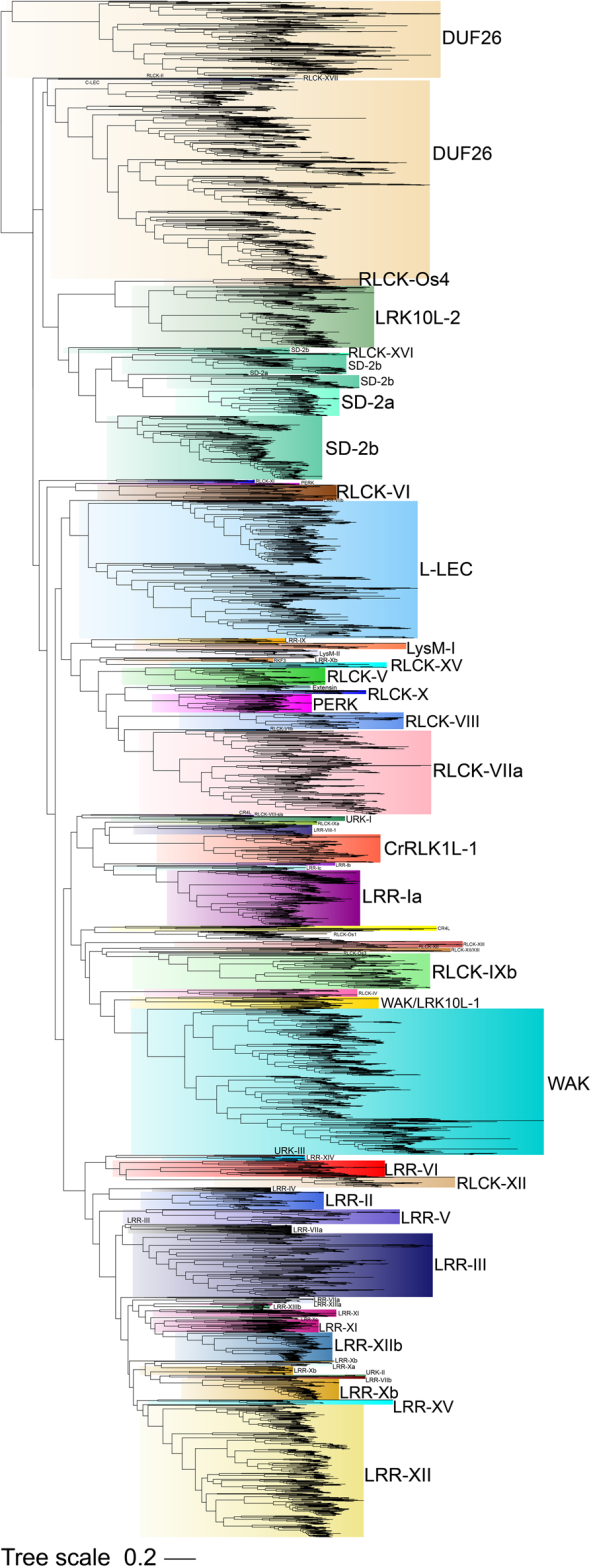
Phylogenetic analysis of *RLKs* in wheat, rice and Arabidopsis. The protein sequences of 3,424 TaRLKs, 613 AtRLKs and 1,087 OsRLKs were aligned using MAFFT with the L-INS-I strategy. The maximum-likelihood phylogenetic tree was constructed using Fasttree with the CAT model (category approximation of GAMMA model of rate heterogeneity) and the Jones-Taylor-Thornton substitution matrix

Protein sequences of 613 AtRLKs from Arabidopsis and 1,087 from rice (*Oryza sativa*) were used as seed sequences to search a local protein kinase database constructed with results from a hidden Markov model search. We finally identified 3,424 *TaRLKs* in wheat, which were classified into 58 subfamilies (Fig. 1, Fig. S1 and Table S1). Analysis of physicochemical properties showed that the amino acid lengths of TaRLKs ranged from 191 aa (TraesCS3D02G013700.1) to 1,716 aa (TraesCS7B02G353800.1), with corresponding molecular weight ranging from 21.15 kDa to 194.18 kDa. The majority (95%, 3,286 genes) had amino acid lengths between 300 and 1,200 aa. The isoelectric points ranged from 4.64 (TraesCS1D02G286800.1 and TraesCS3D02G156400.1) to 11.05 (TraesCS3B02G214300.1). 82% TaRLKs (2,794 genes) exhibited negative average hydropathicity values (Table S1).

**Fig. 2:**
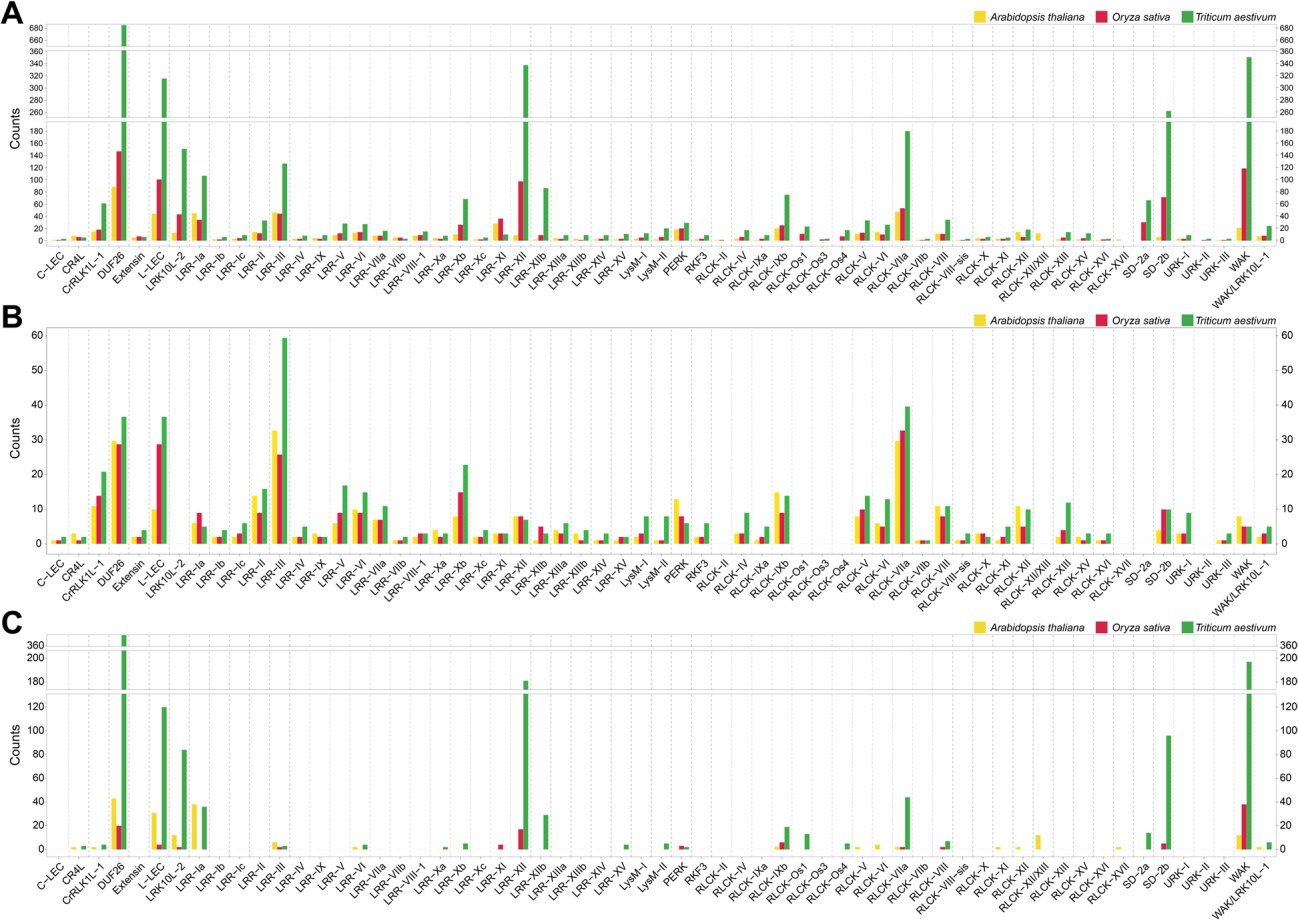
The expansion pattern of *RLKs* subfamily in wheat, rice and Arabidopsis. **A** Comparison of the number of receptor-like kinase subfamily genes in wheat, rice and Arabidopsis. **B** Quantitative distribution patterns of conserved orthologous genes in wheat, rice and Arabidopsis. **C** Quantitative distribution patterns of species-specific expanded orthologous genes in wheat, rice and Arabidopsis

The number of *TaRLKs* identified in wheat was nearly six times that in *Arabidopsis* and more than three times that in rice. Among these subfamilies, the LRR subfamily represented the largest group in wheat, including 937 genes in 22 clades, followed by the RLCK subfamily with 480 genes in 18 clades. Although the DUF subfamily lacked detailed classification, it included 683 genes. In contrast, the C-LEC subfamily contained only three genes (Table S1). Notably, the expansion of subfamily members such as DUF26, L-LEC, LRK10L-2, LRR-XII, LRR-XIIb, RLCK-VIIa and SD-2b exceeded what would be expected solely from chromosome polyploidization. (Fig. 2A).

To investigate the diversity and evolutionary conservation of the RLK gene family among eudicots (Arabidopsis thaliana) and monocots (rice and wheat), we performed orthologous group analysis (Table S2). The results suggested that most subfamilies were conserved during the evolution of Arabidopsis, rice and wheat, though some subfamilies exhibited species-specific expansions. A total of 176 common orthologous groups containing 1,149 RLKs were identified among the three species, (Fig. 2B and Table S2). Of these, 12 groups were strictly one-to-one orthologs, while the remaining 164 groups showed one-to-many, many-to-one or many-to-many orthologous relationships. It suggests that these genes are conserved in evolution and are accompanied by different degrees of expansion, which may have a major contribution to maintaining the unique functions of their subfamilies. Additionally, 37, 30 and 351 species-specific orthologous groups were identified in Arabidopsis, rice and wheat, containing 178, 105 and 1,252 genes, respectively (Fig. 2C and Table S2). In wheat, specific expansion of the LRK10L-2, LRR-XII, RLCK-VIIa, SD-2b and WAK subfamily genes far exceeded that of Arabidopsis and rice, with increases ranging from several-fold to dozens of times. Robust evidence from various plants suggests that these subfamily expansions play crucial roles in response to biotic and abiotic stresses. These findings highlight that the distinct expansion patterns of the TaRLKs subfamily. Conservatively evolved orthologous groups likely maintain the original functions of respective subfamilies, while species-specific extended orthologous groups may contribute significantly to numerical differences among wheat, Arabidopsis and rice and adapt to complex stress signals.

### Selection pressure analysis and conserved domain characteristics of *TaRLKs*

**Fig. 3:**
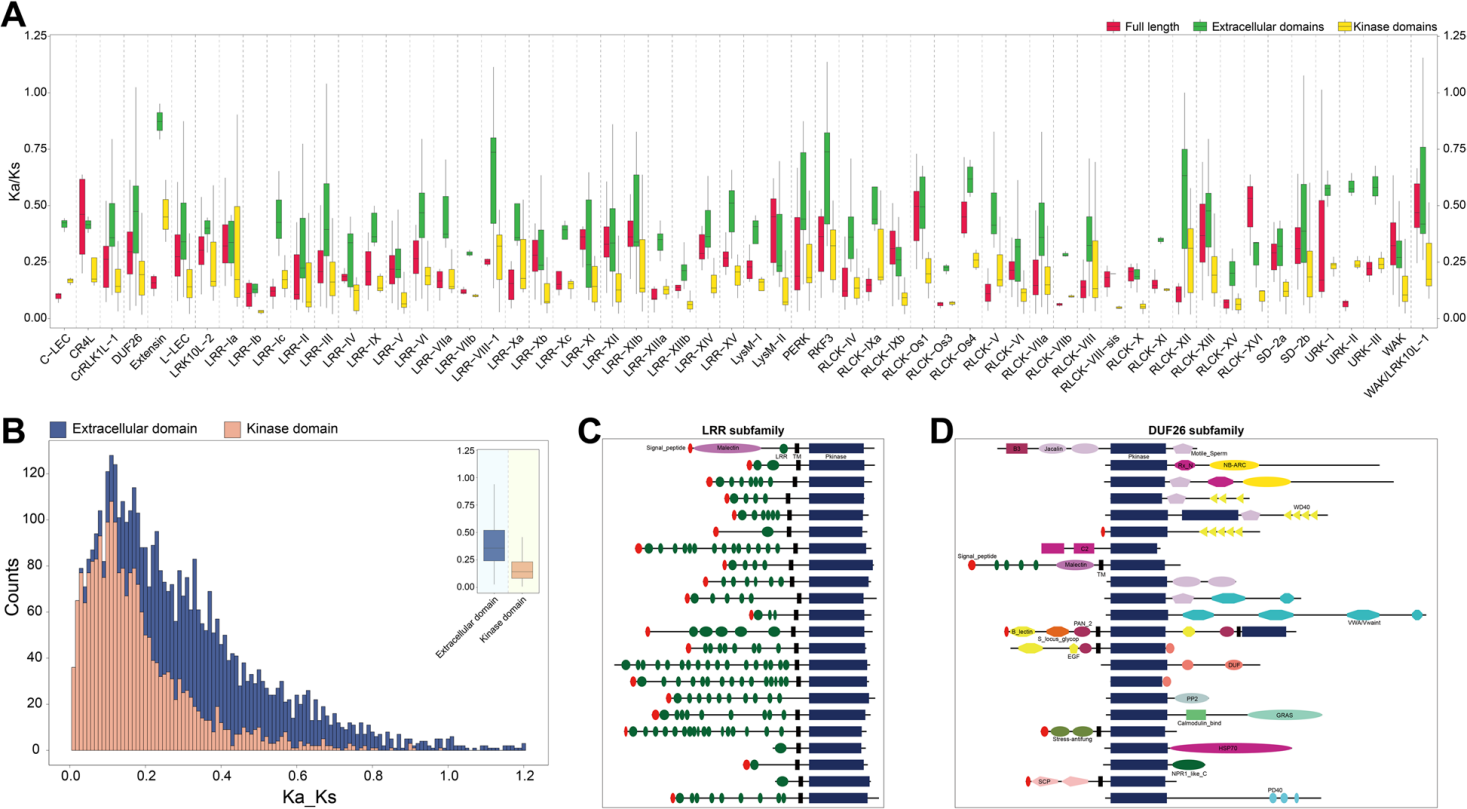
Analysis of selective pressures and conserved domain characteristics in *TaRLKs*. **A** Effects of tandem repeat events on the functional differentiation of the *TaRLKs* subfamily. The y-axis represents the rate of non synonymous (Ka) and synonymous (Ks) replacement (Ka/Ks). **B** The Ka/Ks frequency distribution for extracellular and kinase domains. **C** and **D** Domain characteristics of the LRR (**C**) and DUF26 (**D**) subfamilies, respectively. The same color block represents the same conserved domain, and the same domain is only marked once with text to make the picture concise. Domain names are assigned according to the SMART, NCBI-CDD and Pfam databases

Gene duplication and natural selection play pivotal roles in shaping the diversity of gene function. Given the significant impact of duplication events on the expansion of *RLKs* in plant, we used Ka/Ks ratios to assess the selection pressure of duplicated gene pairs. A total of 2,298 duplicated gene pairs were identified in wheat, with the majority exhibiting Ka/Ks values below 0.5. This suggests that these genes are under purifying selection to maintain important biological function (Fig. 3A and Table S3). Notably, the median and dispersion of Ka/Ks values for *TaRLKs* with extracellular domains (ECDs) were higher than those for *TaRLKs* containing only intracellular kinase domains. Additionally, within the same subfamily, the Ka/Ks values for the ECDs were generally larger than those of the kinase domains (Fig. 3A). The distribution of Ka/Ks ratios further confirmed that ECDs exhibited significantly higher average values compared to kinase domains (Fig. 3B). These findings suggest that ECDs may have undergone weaker purifying selection or stronger diversifying selection, thus evolving new domain features to recognize various extracellular signals. By contrast, kinase domains, constrained by their role in downstream signal transduction, appear to have experienced stronger purifying selection.

The TaRLKs family exhibits a rich diversity in conserved domain characteristics. Genes within the same subfamily generally share similar domain architectures, while different subfamilies have evolved unique structural features to adapt specific functional requirements (Fig. 3C-D and Fig. S1). For example, the LRR subfamily displays variability in the number of conserved LRR motifs within the extracellular ligand recognition region, likely to recognize signaling molecules of differing lengths or types (Fig. 3C). This dynamic modification of extracellular ligand-binding domains is observed across nearly all subfamilies, including WAK, LRK10L-2, SD, L-LEC, and CrRLK1L (Fig. S1). The RLCK subfamily predominantly consists of genes with conserved kinase domains; however, some members include fused domains at one or both ends of the kinase domain (Fig. S1). Meanwhile, the DUF26 subfamily is exceptionally diverse, encompassing nearly all conserved domains present in TaRLKs (Fig. S1).

A few noteworthy exceptions highlight the structural complexity of this gene family. For instance, TraesCS5B02G154600.2 within the LRR subfamily contains both an LRR motif and a Malectin-like domain in its extracellular region (Fig. 3C). TraesCS5D02G357700.1 appears to form a unique RLK with two kinase domains, two transmembrane domains, and two potential ligand-binding domains, while TraesCS6B02G029600.1 contains only two kinase domains (Fig. S1). In addition, Traescs5B02G534000.2 in the DUF26 subfamily possesses four repeated VWA/Vwaint domains at the C-terminus of its kinase domain, potentially making it an oversized RLCK (Fig. 3D). These findings highlight the structural and functional diversity of the large TaRLKs family. The continuous emergence of novel structural features suggests that TaRLKs evolve through diverse mechanisms to recognize and respond to various signals, ensuring their adaptability to environmental challenges.

### Conserved motif analysis of ATP binding site and kinase active site

Protein kinases play an essential regulatory role in numerous biological processes. To investigate conserved motifs within the kinase domain that may influence kinase activity, we conducted multiple sequence alignments of 3,424 identified TaRLKs and visualized 80 key sites near possible ATP binding sites and kinase active sites. The analysis suggested that 94.7% (3,244/3,424) of TaRLKs harbor a conserved ATP-binding site lysine (K) within the motif [V/I-A-V/I-K] (Fig. S2A), and 91.1% (3,120/3,424) possess a conserved kinase active site aspartate (D) in the motif [xxHxDxKxxNxLLD] (Fig. S2B). These findings suggest that these conserved amino acids have undergone strong purifying selection. Histidine within [xxHxDxKxxNxLLD] motif is critical for forming a hydrogen-bond network that maintains the structural integrity of the kinase catalytic center (Zhang et al. 2015). Similarly, the conserved residues K, N, L, and D are likely to play crucial roles in regulating kinase activity and ensuring proper enzymatic function.

### Conserved constitutively highly expressed *RLKs* in wheat, Arabidopsis and rice

**Fig. 4:**
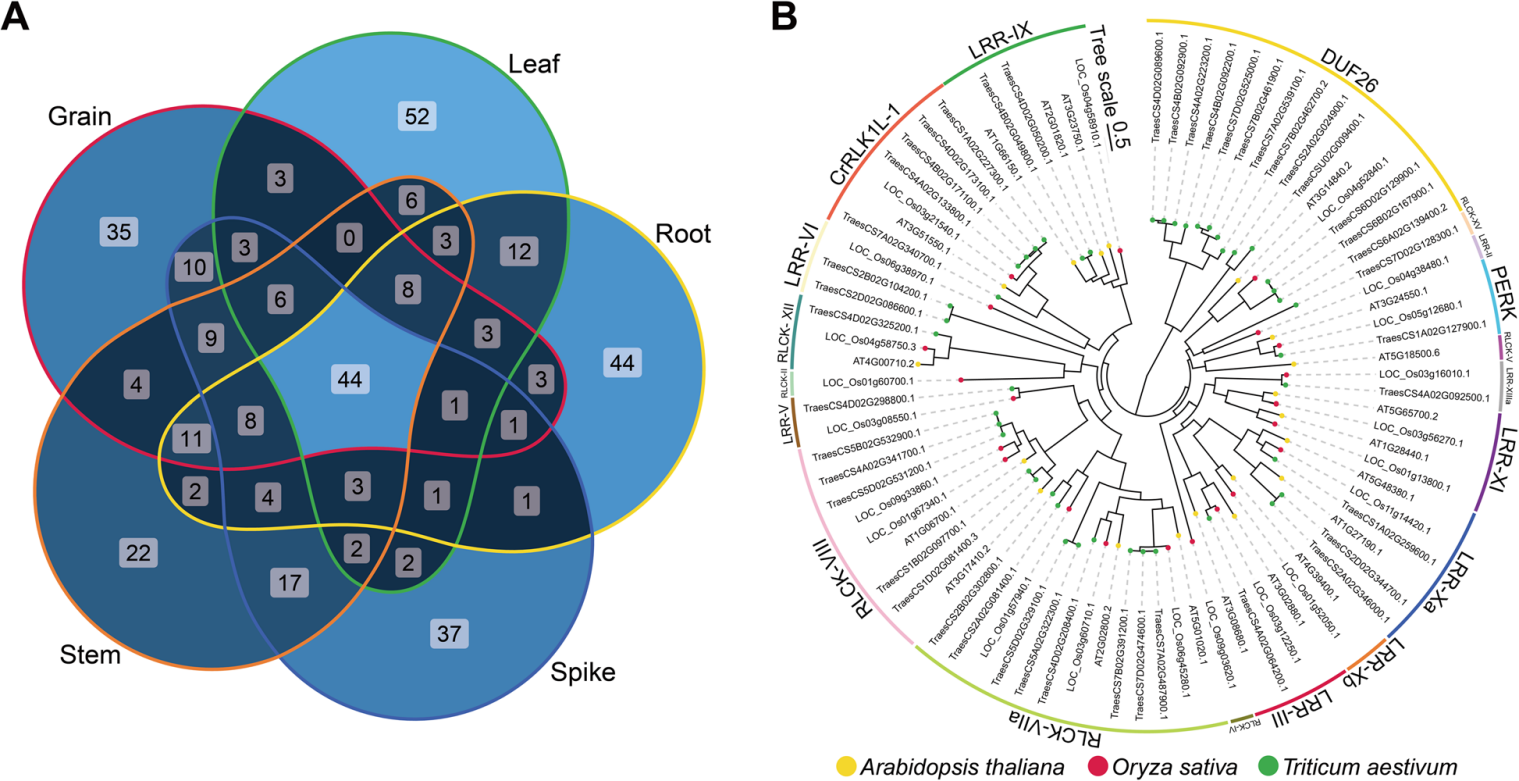
Convergent evolution of constitutive highly expressed *RLKs* in root, spike, stem, grain and leaf. **A** Expression intersection of *TaRLKs* in root, spike, stem, grain and leaf. **B** Phylogenetic analysis of constitutively highly expressed *RLKs* in Arabidopsis, rice and wheat

In order to investigate the expression patterns of *TaRLK* family members in different wheat tissues, we analyzed the publicly available wheat gene expression data (Table S5). Interestingly, among the 150 most highly expressed *TaRLKs* in leaf, root, spike, stem and grain, 44 were commonly expressed across all tissues. These include 5 CrRLK1Ls, 13 DUF26s, 10 LRRs, 15 RLCKs and 1 PERKs (Fig. 4A). Similar findings have been reported in rice and Arabidopsis (Shiu and Bleecker 2001; Shiu et al. 2004), including one CrRLK1L, 11 LRRs, one PERK and 6 RLCKs in Arabidopsis, as well as 1 CrRLK1L, 11 LRRs, 1 PERK and 8 RLCKs in rice. Interestingly, some of these constitutively highly expressed genes are highly conserved during evolution (Fig. 4B). Members of the PERK, LRR-Xa, LRR-III, LRR-XI, RLCK-VIIa, RLCK-VIII, RLCK-XII, LRR-IX and CrRLK1L-1 subfamilies, which are highly expressed in Arabidopsis and rice, are homologous to the corresponding highly expressed genes in wheat. This suggests that these genes likely originated before the divergence of monocots and dicots and play fundamental and conserved roles in plant development. Notably, the 13 DUF26 subfamily genes identified in this study represent a newly observed class of constitutively highly expressed genes in wheat. These genes may have specialized roles in wheat growth and development.

### Expression patterns of *TaRLKs* in wheat-*Pst* interaction

**Fig. 5:**
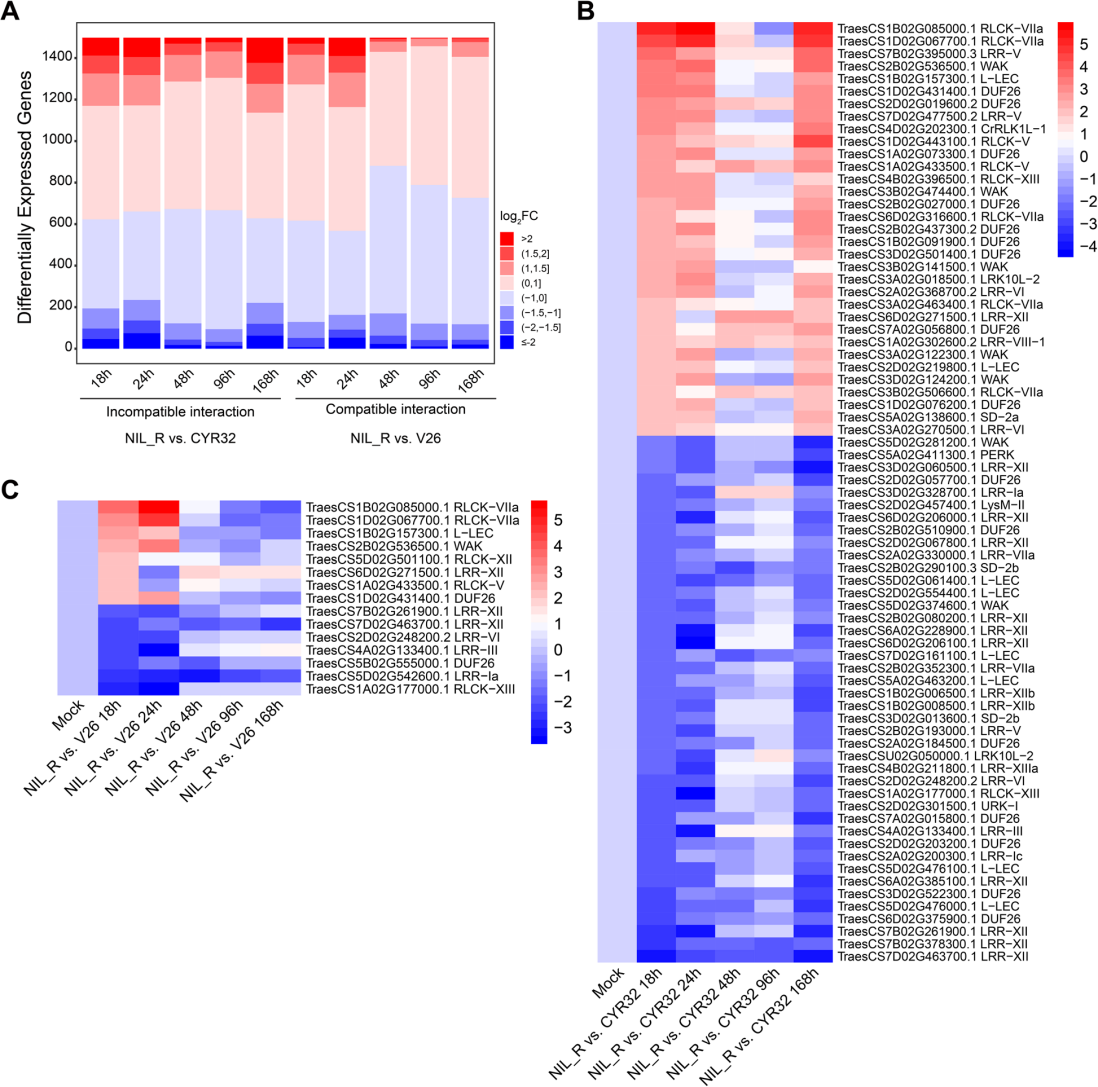
Differentially expressed *TaRLKs* in compatible and incompatible interaction between wheat and *Pst*. **A** The accumulation map of differentially expressed *TaRLKs* in incompatible interaction (NIL_R vs. CYR32, **left**) and compatible interaction (NIL_R vs. V26, **right**) at different time points. The related logarithmic fold change in expression is represented by a gradient from blue to red, where blue represents downregulation and red represents upregulation. **B** Heatmap of differentially expressed *TaRLKs* in the incompatible group at 18 hpi. **C** Heatmap of differentially expressed *TaRLKs* in the compatible group at 18 hpi. Only genes with TPM values greater than 0.5 in the mock group and absolute values of log_2_FC greater than 2 at 18 hpi are displayed in **B** and **C**

To investigate the potential function of *TaRLKs* in wheat-*Pst* interactions, we analyzed the transcription level of *TaRLKs* after inoculation with compatible and incompatible *Pst* races (Table S4). Differential expression analysis showed that many *TaRLKs* were significantly up- or down-regulated in response to *Pst* (Fig. 5A), suggesting their involvement in the host response to *Pst*. To further explore the potential functions of *TaRLKs*, we visualized *TaRLKs* with a baseline expression level greater than 0.5 and a significant fold change (|log_2_FC| ≥ 2) at 18 h post-inoculation (hpi) with *Pst* (Fig. 5B and C). In the incompatible interaction group (Fig. 5B), significantly induced *TaRLKs* were primarily clustered within the DUF26, LRR-XII, L-LEC, WAK, and RLCK-VIIa subfamilies. Growing evidences suggests that members of these subfamilies are commonly involved in plant responses to various stresses. These significantly induced genes should be focused for subsequent functional verification. In the compatible interaction group (Fig. 5C), significantly induced *TaRLKs* were concentrated within the LRR-XII, RLCK-VIIa and DUF26 subfamilies. Interestingly, 12 *TaRLKs* were differentially expressed in both compatible and incompatible interaction, suggesting that these genes may play multiple roles in regulating wheat responses to *Pst* infection. In summary, numerous *TaRLKs* were significantly differentially expressed after inoculation with *Pst*, suggesting their potential importance in the wheat defense response to *Pst*.

### Expression patterns of *TaRLKs* in response to abiotic and biotic stresses

**Fig. 6:**
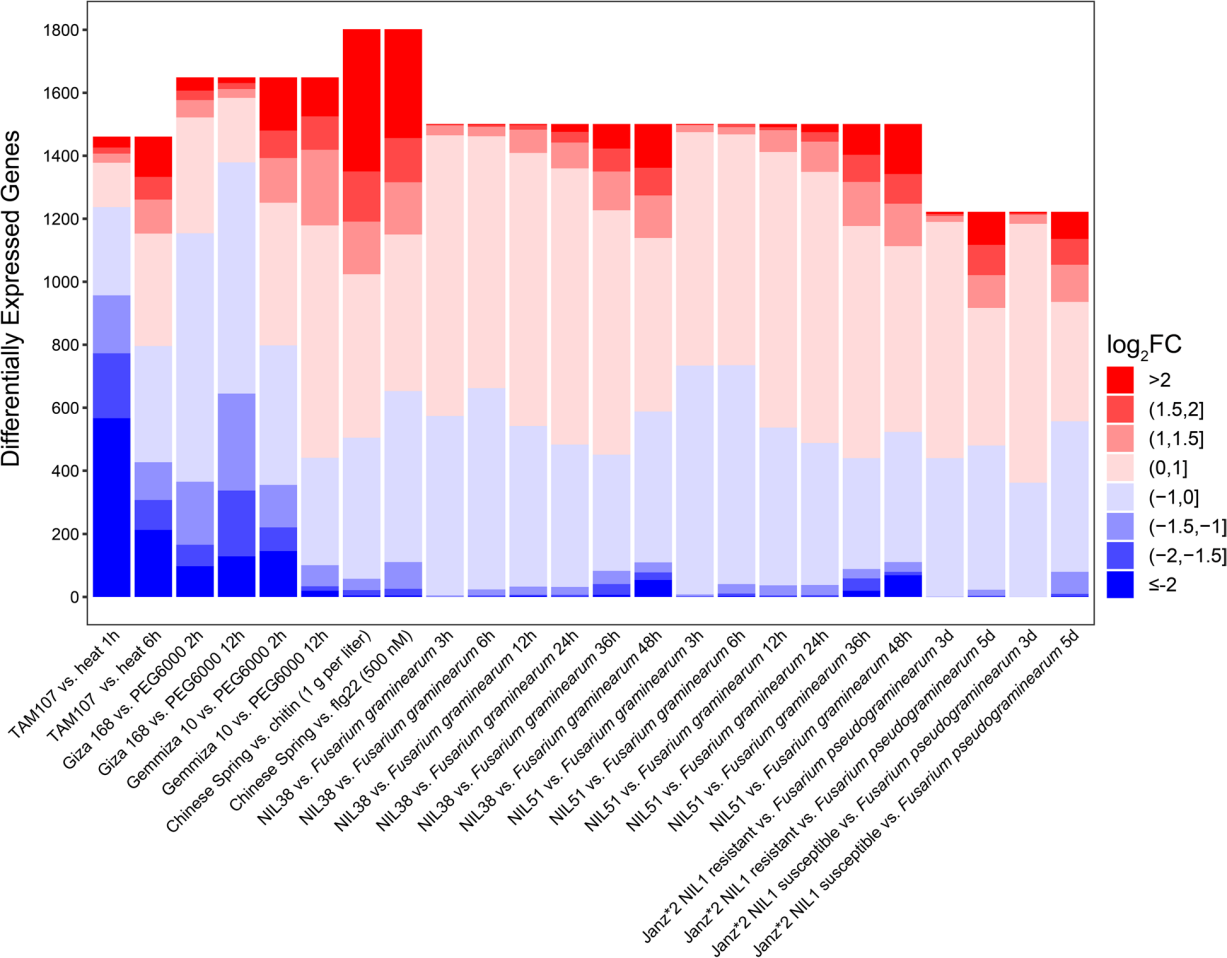
The accumulation map of differentially expressed *TaRLKs* in response to biotic and abiotic stresses

We further analyzed the expression patterns of *TaRLKs* in response to various stresses, including chitin (1 g/L), flg22 (500 nM), heat, PEG6000, *F. graminearum*, and *F. pseudograminearum* using public data from the expVIP database (Table S5). Genes with undetectable transcript levels were excluded from further analysis. The results suggested that many *TaRLKs* seem to be involved in responses to both abiotic and biotic stresses (Fig. 6). To provide a clearer view of stress-responsive *TaRLKs*, transcriptome data were filtered using a higher threshold (TPM values > 0.5 in control conditions and the |Log_2_FC|> 2). Notably, a large number of *TaRLKs* were significantly induced following stimulation with flg22 and chitin, with the number of upregulated genes greatly exceeding downregulated genes (Fig. 6). Among these, 138 *TaRLKs* were co-induced by flg22 and chitin (Fig. S3C). In response to heat stress, the majority of *TaRLKs* were downregulated at one hour post treatment (hpt), but the pattern shifted at 6 hpt, with a significant increase in upregulated genes and fewer downregulated ones (Fig. 6). It is noteworthy that 139 *TaRLKs* showed significant differential expression at both time points (Fig. S3A). Under drought stress, contrasting expression patterns were observed in two wheat varieties: Giza 168 (drought-tolerant) displayed more downregulated *TaRLKs*, whereas Gimmeza 10 (drought-sensitive) exhibited more upregulated genes (Fig. 6). Specifically, opposing gene expression patterns were noted after 2 h of drought stress in these two varieties (Fig. S3B). For infection with *F. graminearum*, the number of differentially expressed *TaRLKs* increased over time in both NIL 38 (carrying QTL *Fhb1* and *Qfhs.ifa-5A*) and NIL 51 (lacking these QTLs), with highly consistent expression patterns between the two genotypes (Fig. S3E). Furthermore, during *F. pseudograminearum* infection, the number of upregulated *TaRLKs* at 5 days post-infection was several times higher than at 3 days (Fig. S3D). These findings suggest that *TaRLKs* play key roles in responses to diverse abiotic and biotic stresses.

### Expression analysis of CrRLK1L subfamily members and functional characterization of *TaCrRLK1L16* in wheat-*Pst* interaction

**Fig. 7:**
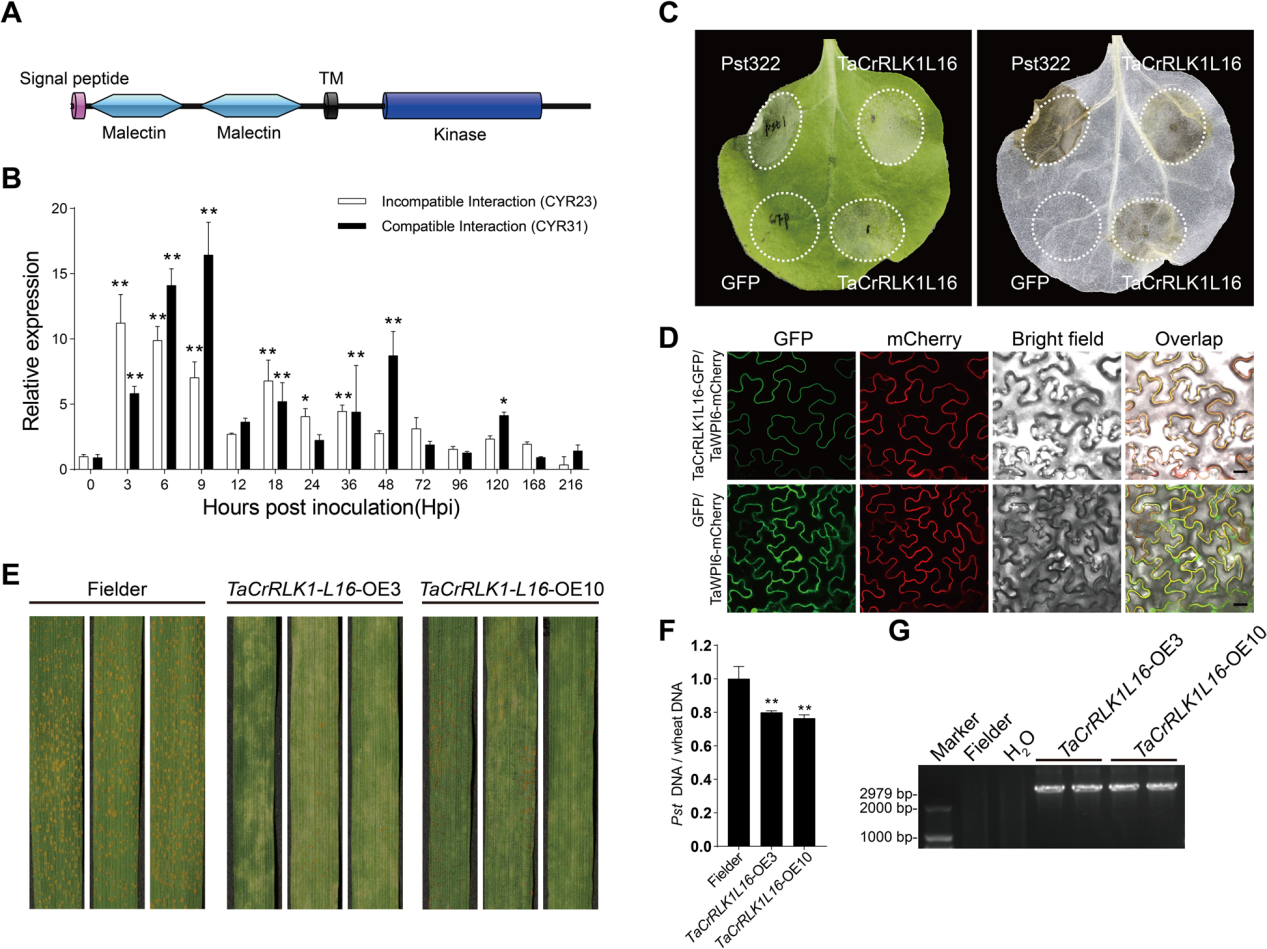
Functional characterization of *TaCrRLK1L16* in the wheat-*Pst* interaction. **A** Schematic representation of the conserved domains in the TaCrRLK1L16 protein. **B** Transcript levels of *TaCrRLK1L16* in wheat leaves were induced during *Pst* infection (*p < 0.05, ** *p* < 0.01). **C** Transient overexpression of *TaCrRLK1L16* triggered cell death in *N. benthamiana*. Pst322 and GFP were used as positive and negative control, respectively. **D** Subcellular localization of *TaCrRLK1L16* in *N. benthamiana*. TaWPI6-mCherry was employed as a plasma membrane marker.** E** Phenotypic analysis of *TaCrRLK1L16*-OE3 and *TaCrRLK1L16*-OE10 transgenic wheat lines inoculated with *Pst* CYR34. **F** Biomass ratio (fungal to wheat) determined using the DNA content of fungal *PstEF1* and wheat *TaEF-1α* genes. Genomic DNA was extracted from three different plants at 7 days post-inoculation. Error bars represent variation among three biological replicates, and asterisks indicate significant differences between that in transgenic and Fielder plants at the same time points (**p < .01, Student's *t*-test). **G** Detection of two independent T1 positive transgenic lines (*TaCrRLK1L16*-OE3 and *TaCrRLK1L16*-OE10) by PCR

Plant immunity is closely associated with the HR (Pitsili et al. 2020). Recent studies on *RLKs* positively regulating plant immunity suggested that transient overexpression of *RLKs* can trigger cell death in *N. benthamiana* (Zhang et al. 2024). In order to quickly determine the priority of key candidate genes for functional analysis, we cloned some candidate *TaRLKs* induced by *Pst* from wheat cultivar Fielder. Priority was assigned based on the extent of HR observed following transient overexpression of *TaRLKs* in *N. benthamiana*. Among them, transient overexpression of TraesCS4D02G202300.1 (identified in Fig. 5B) triggered cell death comparable to the positive control, *Pst322* (an elicitor-like protein from *Pst*), suggesting its potential role in activating plant defense (Fig. 7C). Consequently, *TraesCS4D02G202300.1* was selected for further functional characterization.

TraesCS4D02G202300.1 belongs to the CrRLK1L subfamily. CrRLK1L plays an important role in plant growth, and development and stress response, while its role in resistance to *Pst* has been minimally studied. Phylogenetic analysis divided the wheat CrRLK1L subfamily into six groups (Group I-VI, Fig. S4), naming 28 members (*TaCrRLK1L1-TaCrRLK1L28*; Table S6). Transcriptome data for the incompatible interaction (NIL_R vs. CYR32) suggested that *TaCrRLK1L16* exhibited the highest expression level within Group I (Fig. S6). Three highly similar alleles of *TaCrRLK1L16* were identified on chromosomes 4A (*TaCrRLK1L16-4A*, TraesCS4A02G102900.1), 4B (*TaCrRLK1L16-4B*, TraesCS4B02G201600.1), and 4D (*TaCrRLK1L16-4D*, TraesCS4D02G202300.1) (Fig. S5). NCBI BLAST analysis showed that TaCrRLK16 shares high sequence similarity with HERK in Arabidopsis and OsCrRLK17 in rice, both of which contain an N-terminal signal peptide, two extracellular Malectin-like domains, a single transmembrane domain and an intracellular kinase domain (Fig. 7A and Fig. S5). The sequence similarity suggests potential conserved functional roles.

In this study, functional analysis focused on *TaCrRLK1L16-4D* due to its high sequence similarity with its alleles. *TaCrRLK1L16-4D* has an open reading frame (ORF) of 2,550 bp, encoding a protein of 849 amino acids. Quantitative PCR confirmed the transcriptomic results, showing that *TaCrRLK1L16* expression was significantly upregulated during *Pst* infection, peaking at 3 hpi and 9 hpi in incompatible and compatible interactions, respectively (Fig. 7B). Subcellular localization analysis in *N. benthamiana* co-expressing *TaCrRLK1L16-GFP* with a plasma membrane marker (*TaWPI6-mCherry*) suggested overlapping GFP and mCherry signals, indicating that *TaCrRLK1L16* localizes to the plasma membrane (Fig. 7D).

To validate the functional role of *TaCrRLK1L16* in wheat resistance to *Pst*, transgenic wheat lines overexpressing *TaCrRLK1L16* (*TaCrRLK1L16-OE*) were developed using cultivar Fielder as the receptor plant. PCR confirmed two independent T1 positive transgenic lines (*TaCrRLK1L16*-OE3 and *TaCrRLK1L16*-OE10; Fig. 7G). Infection assays with *Pst* race CYR34 suggested significantly reduced urediniospore production and fungal biomass in *TaCrRLK1L16-OE* plants compared to non-transgenic controls (Fig. 7E and F). These results suggest that elevated expression of *TaCrRLK1L16* enhances wheat resistance to *Pst*.

## Discussion

Plant RLKs play a pivotal role in recognizing various signals and activating intracellular immune responses (Jones and Dangl 2006; Tian et al. 2021). The first putative plant receptor protein kinase gene, ZmPK1, was cloned and characterized by Walker and Zhang in 1990 (Walker and Zhang 1990). To date, the RLKs family has been identified in hundreds of plant species (Ngou et al. 2022; Liu et al. 2024a, b; Yin et al. 2024). In this study, we identified 3,424 *TaRLKs*, which were divided into 58 subfamilies. The total number of predicted genes in wheat is approximately ten times and five times greater than that in Arabidopsis and rice, respectively, and the *TaRLKs* gene count is almost six and three times that of Arabidopsis and rice, respectively. This expansion is likely attributed to the larger genome size of hexaploid wheat.

Despite the substantial variation in the number of RLK family genes across different species, it is noteworthy that 176 orthologous groups spanning 52 subfamilies were identified in Arabidopsis, rice, and wheat. These conserved evolutionary subfamily genes may be sufficient to retain their original biological functions through chromosome polyploidization. Subfamilies such as C-LEC, LRR-Ib, LysM-I, RLCK-XIII and URK-I seemed to have no additional amplification except for chromosome polyploidization. It seems that there are only one or two copies of C-LEC in most plants, and its function remains a mystery (Dievart et al. 2020). In the dicot model plant Arabidopsis, the lysin motif (LysM)-containing chitin elicitor receptor kinase 1 (CERK1) has been shown to be essential for perception of the fungal cell wall component chitin and for resistance to fungal pathogens (Petutschnig et al. 2010). Chitin elicitor receptor kinase 1 of *Oryza sativa* (OsCERK1) plays a bifunctional role in mediating both chitin-triggered immunity and symbiotic relationships with AM fungi. Recent studies have shown that OsCERK1 binds to the chitin hexamer ((NAG)_6_) and tetramer ((NAG)_4_) directly (Xu et al. 2023). These subfamilies may maintain ancient and conserved multiple biological functions.

However, the genes numbers across subfamilies exhibit considerable variation. Compared with rice and Arabidopsis, the expansion of DUF26, L-LEC, LRK10L-2, LRR-XII, LRR-XIIb, RLCK-VIIa and SD-2b subfamilies in wheat obviously exceeded the number difference caused by chromosome polyploidization. Remarkably, we identified 37, 30, and 351 species-specific orthologous groups in Arabidopsis, rice, and wheat, containing 178, 105, and 1,252 genes, respectively (Fig. 2C). The specific expansion of LRK10L-2, LRR-XII, RLCK-VIIa, SD-2b and WAK subfamily genes in wheat ranging from several- to dozens-fold that in Arabidopsis and rice likely reflects selective pressures from biotic stress. suggest that this may also be an important reason for the difference in the number of RLKs family genes. These subfamilies are often implicated in stress responses. For instance, LRR-XII subfamily member FLS2 and EFR recognize bacterial flagellin and elongation factor Tu (EF-Tu), respectively, activating immune defenses (Gómez-Gómez and Boller 2000; Zipfel et al. 2006). RLCK VII subfamily members, such as BIK1, play critical roles in PTI, phosphorylating RBOHD to regulate ROS bursts and calcium influx (Kadota et al. 2014; Tian et al. 2019). Monocot-specific RLK SDS2, an S-domain family (SD-1a type) RLK, regulates cell death and immunity in rice (Fan et al. 2018). The specific expansion of defense-related RLKs likely reflects adaptation to pathogen pressures.

Most *TaRLKs* exhibit evidence of purifying selection (Ka/Ka < 1), with most Ka/Ks ratios below 0.5. RLKs with extracellular ligand-binding domains show higher non-synonymous mutation rates compared to RLCKs with only intracellular domain. Moreover, the extracellular domains usually have higher non-synonymous mutation rates than intracellular kinase domains. The results suggest that extracellular domains have undergone weak purification or strong diversification selection to recognize various signals, while intracellular kinase domains have experienced stronger purifying selection to ensure activation of similar cellular signal transduction. Despite the rich diversity of domain architectures in *TaRLKs*, the limited conserved domains appear insufficient to recognize the diversity of novel “self” or “non-self” ligands. Some RLKs form novel domain architectures by fusing or rearranging domains (Fig. S1). For example, TraesCS5D02G357700 appears to consist of two complete RLKs in tandem, with its function yet to be elucidated. Other RLK members, such as TraesCS6B02G029600, TraesCS1B02G079900, TraesCS7B02G423700 and TraesCS3B02G578100, feature dual kinase domains, potentially serving as a protective mechanism against pathogen interference.

Shiu et al. reported constitutively highly expressed RLKs in rice and Arabidopsis, mainly distributed in the LRR, RLCK, CrRLK1L and PERK subfamilies (Shiu and Bleecker 2001; Shiu et al. 2004). Similarly, we identified 44 overlaps among the 150 *TaRLKs* with the highest expression levels in wheat leaves, roots, spikes, stems and grains, including five CrRLK1Ls, 13 DUFs, 10 LRRs, 15 RLCKs and one PERK. Among these, 13 DUF26 genes represent newly identified highly expressed genes, likely playing conservative and fundamental roles in wheat growth and development.

Transcriptome analysis suggest that many *TaRLKs* are strongly induced under biotic and abiotic stresses. Significantly induced *TaRLKs* are clustered in the L-LEC, LRR and WAK subfamilies, and increasing evidences showed that these subfamily members are widely involved in stress response. These significantly induced genes should be focused on in subsequent functional verification. In addition, in the future in-depth study, the significantly induced *TaRLKs* at different time points should not be ignored. However, only a few functions of *TaRLKs* have been studied in response to biotic stresses in plants. Even with new paradigms such as FER, functional studies in wheat are still limited. As the largest gene family in plants, many unstudied members may also be involved in important central roles in balancing plant immunity and growth and development.

Multi-omics association analysis provides data support for the study of plant gene function, but the functional characterization of large gene families still faces difficulties in anchoring key candidate genes. An effective research strategy is crucial to characterize the function of *TaRLKs*. In this study, we identified *TaRLK1L16* as a significantly upregulated gene induced by *Pst* through transcriptome analysis. Functional assays demonstrated that heterologous expression of *TaRLK1L16* triggered cell death in *N. benthamiana*, while transgenic wheat lines overexpressing *TaRLK1L16* showed enhanced resistance to *Pst* infection. As a plasma membrane-localized *RLK*, *TaCrRLK1L16* may recognize unknown extracellular ligands, thereby initiating conserved intracellular immune pathways, and produce a variety of defense outputs to resist the *Pst* infection. At present, there are few studies on the function of immune receptors in wheat and other food crops, so there is a lack of sufficient evidence to analyze the recognition mechanism of receptor-like kinases in food crops to signal molecules and the activation mechanism of immune signals. In this study, the differentially expressed genes were screened by transcriptome data analysis and transiently expressed in *N. benthamiana* to preliminarily clarify their basic functions, and then their biological functions were tested by transgenic plants. This research strategy will greatly improve the rapid characterization of the function of plant receptor-like kinase family genes.

Wheat contributes approximately one-fifth of global caloric intake and serves as the leading source of dietary protein worldwide. However, devastating diseases such as stripe rust (caused by *Pst*) significantly threaten wheat yield. The rapid evolution of *Pst* often overcomes existing resistance in wheat cultivars, leading to disease pandemics. Moreover, due to the lack of rust resistance gene resources, the speed of rust resistance breeding lags behind pathogen variation. The cultivation of broad-spectrum and durable disease resistant wheat varieties is facing serious challenges, underscoring the urgent need to identify novel immune receptor gene resources.

## Conclusions

In summary, we identified 3,424 *TaRLKs* in wheat, classifying them into 58 subfamilies. While most subfamilies have evolved conservatively to maintain their ancestral biological functions, some species-specific expanded subfamilies may have crucial roles in defending against diverse pathogenic microorganisms. The extracellular domains of *TaRLKs* exhibit continuous evolution to recognize various signaling molecules, whereas the intracellular kinase domains are under strong purifying selection to preserve their role in activating conserved signal nodes. *TaRLKs* are induced by various biotic and abiotic stresses, including *Pst*. In this study, we proposed a strategy to determine the priority of candidate genes identified through transcriptome analysis based on the HR phenotype in tobacco, and confirmed that the elevated expression of *TaCrRLK1L16* enhanced the wheat resistance to *Pst* infection. In the future, integrating multi-omics data with multi-species evolutionary analyses and utilizing transient overexpression systems in tobacco to rapidly characterize the fundamental biological functions of candidate genes will streamline the determine the experimental priority. This strategy promises to accelerate reverse genetics research on large gene families, such as *TaRLKs*, in wheat and other gramineous crops.

## Meterials and methods

### Whole genome identification of *TaRLKs* in wheat

To identify putative protein kinases in the wheat genome, Hidden Markov Models of the Pkinase domain (PF00069) and the Pkinase-Tyr domain (PF07714) were employed to search the wheat whole genome protein sequences (IWGSC RefSeq v1.1 http://ftp.ebi.ac.uk/ensemblgenomes) using HMMER (Ramírez-González et al. 2018). RLK protein sequences of Arabidopsis and rice were used as seed sequences to perform local BLASTP searches against a custom protein database generated from the identified putative protein sequences (Shiu and Bleecker 2001; Shiu et al. 2004). The conserved domains of all candidate *TaRLKs* sequences were annotated using SMART, Pfam and NCBI-CDD databases (Lu et al. 2019; Letunic et al. 2020; Mistry et al. 2020). Subsequently, all RLK protein sequences of Arabidopsis, rice, and wheat were used to generate multiple sequence alignments using L-INS-I strategy with MAFFT (Katoh and Standley 2013). The maximum-likelihood phylogenetic tree was constructed using FastTree with the CAT model (category approximation of GAMMA model of rate heterogeneity) and the JTT (Jones, Taylor, Thornton) substitution matrix (Price et al. 2010). Sequences lacking a kinase domain or containing incomplete kinase domains were filtered out. The phylogenetic trees and conserved domains were visualized using iTOL and TVBOT (Letunic and Bork 2021; Xie et al. 2023). Protein sequences of AtCrRLK1Ls, OsCrRLK1Ls, and TaCrRLK1Ls were aligned using MAFFT with L-INS-I strategy (Cheung and Wu 2011; Nguyen et al. 2014). The phylogenetic tree of CrRLK1L subfamily was constructed using the maximum likelihood method with IQ-TREE 2 based on JTT + F + R8 model with 1,000 bootstrap replications (Minh et al. 2020). Signal peptide, transmembrane helix, and subcellular localization were predicted using SignalP-5.0, TMHMM-2.0, and CropPAL, respectively (Krogh et al. 2001; Hooper et al. 2015; Almagro Armenteros et al. 2019). Molecular weight and isoelectric point of proteins were calculated using ExPASy (Gasteiger et al. 2005). ATP binding site and kinase active site sequences were extracted from the NCBI-CD and visualized as sequence logos using TBtools-II, highlighting conserved amino acids (Chen et al. 2023).

### Orthologous groups, gene duplication pairs and selection pressure of *TaRLKs*

Othologous groups of all RLKs in Arabidopsis, rice and wheat were identified using OrthoFinder (Emms and Kelly 2019). Gene duplication pairs were detected using the ‘One step MCScanX’ tool in TBtools-II based on gff3 annotation and genome sequence files (Chen et al. 2023). Additionally, the extracellular and intracellular domain sequences were extracted according to their domain location to calculate the nonsynonymous (Ka) and synonymous (Ks) ratio (Ka/Ks) using the ‘Simple Ka/Ks Calculator’ function in TBtools-II (Chen et al. 2023).

### Gene transcription analysis, fungal pathogens, treatments and plant materials

Transcript levels of *TaRLKs* were analyzed by time series dual RNA-seq data. We sequenced two groups of wheat-rust interaction combination, named NIL_R vs. V26 (compatible group) and NIL_R vs. CYR32 (incompatible group), and selected the time point at 0, 18, 24, 48, 96 and 168 hpi. The specific method is the same as our previous study (Guo et al. 2018). Wheat plants used for molecular analysis were grown in a greenhouse at 25 °C /23 °C day/night temperatures, long-day conditions (16 h light/8 h dark photoperiod). The sequencing data have been submitted to CNSA (https://db.cngb.org/cnsa/) of CNGBdb with accession code CNP0001524 (Bai et al. 2021). *Pst* races CYR23, CYR31 and CYR34 were used in this study. Inoculation procedure is the same as a previous study (Wang et al. 2007).

Suwon 11, carrying the *YrSu* gene, is resistant to CYR23 infection but highly susceptible to CYR31. The second leaves inoculated with CYR23 or CYR31 or treated with sterile distilled water (control) were harvested at 0, 3, 6, 9, 12, 18, 24, 36, 48, 72, 96, 120, 168, and 216 hpi for RNA extraction. Time points were selected based on a previous study (Wang et al. 2007). We used the spring wheat variety Fielder as the background for transgenics because of its high transgenic success rate. Fielder contains *Yr6* and *Yr20* genes and shows a compatible interaction with CYR34.

The expression profile datasets of wheat variety “Chinese Spring” were obtained from the Wheat Expression Browser powered by expVIP (Borrill et al. 2016). According to the gene ID, we searched the expression data of *TaRLKs* under different abiotic and biotic stress conditions (including chitin, flg22, heat, PEG6000, *F. graminearum* and *F. pseudograminearum*). Additionally, the expression level of various tissues was analyzed. Heatmaps were visualized using R packages “pheatmap” (Raivo 2010). The Venn diagram was generated using R packages “ggVennDiagram” (Gao et al. 2021).

### DNA and RNA extraction and RT-qPCR analysis

To assay transcript levels of *TaCrRLK1L16*, leaves of wheat Suwon11 inoculated with CYR23 or CYR31 were sampled at 0, 3, 6, 9, 12, 18, 24, 36, 48, 72, 96, 120, 168 and 264 hpi. Total RNA was extracted with the Quick RNA isolation Kit reagent (Huayueyang Biotechnology, China, Beijing) according to the manufacturer’s instructions. First strand cDNA was synthesized using 3 μg of RNA by RevertAid First-Strand cDNA Synthesis Kit (MNI, K1622) according to the manufacturer’s instructions. RT-qPCR was run on CFX Connect Real-Time System (BioRad, Hercules, CA, USA). The specific primer pairs used are listed in Table S7. Gene expression was quantified with the comparative 2^–△△CT^ method. The Transcript level of *TaCrRLK1L16* was normalized to *TaEF*. Each sample was analyzed with three biological replicates. Each biological replicate comprises three to four plant leaves. Each RT-qPCR analysis included three replications. The statistical significance was evaluated with unpaired two-tailed Student’s t test. Genomic DNA was extracted with CTAB.

### Agro-infiltration assays in *N. benthamiana*

For the *TaCrRLK1L16*-induced cell assay, the *TaCrRLK1L16* sequence was cloned into the pGR106 vector and transformed into *Agrobacterium tumefaciens* strain GV3101. For transient expression in *N. benthamiana*, the GV3101 strain carrying corresponding construct were cultured in LB medium at 28 °C with shaking at 220 rpm for 18 h. Cells were collected by centrifugation at 5,000 × g for 5 min and washed three times with MES buffer (10 mM MgCl_2_, 10 mM MES, 200 μM acetosyringone, pH 5.7). Resuspension was kept in the dark for 3 h at RT and infiltrated into leaves of 4- to 6-wk-old *N. benthamiana* with appropriate concentrations (OD600 = 0.3). Pst322 and GFP were used as positive and negative controls, respectively. The *N. benthamiana* plants were grown in a controlled growth chamber under a 16 h/8 h day/night condition at 22 °C/16 °C.

For the subcellular localization of *TaCrRLK1L16*, the sequence was cloned into the pCAMBIA1300 vector, GV3101 strain carrying the corresponding construct was agroinfiltrated into the 4-week-old *N. benthamiana* leaves according to the same method as above. GFP was used as a control and TaWPI6-mCherry as a plasma membrane marker (Imai et al. 2005). GFP and mCherry signals were observed using FV3000 confocal laser scanning microscope (Olympus, Tokyo, Japan).

### Genetic transformation of wheat

To overexpress *TaCrRLK1L16* in transgenic wheat plants, the *TaCrRLK1L16* coding sequence was cloned into the pCUB vector driven by the maize ubiquitin promoter, and transformed into *A. tumefaciens* strain EHA105. The wheat cultivar ‘Fielder’ was used as the receptor material for generating transgenic plants by *Agrobacterium*-mediated transformation (Hayta et al. 2019). Positive *TaCrRLK1L16*-overexpressing transgenic plants were identified by PCR using primers targeting the Ubi promoter and NOS terminator regions. Phenotypes of the second leaves of T1 transgenic lines were photographed 14 d after inoculation.

## Supplementary Information

Additional file 1: Figure S1. Phylogenetic analysis of RLKs in wheat, rice and Arabidopsis and 3,424 conserved domains of TaRLKs. The protein sequences of 3,424 TaRLKs, 613 AtRLKs and 1,087 OsRLKs were aligned using MAFFT with the L-INS-I strategy. The maximum-likelihood tree was constructed with Fasttree using the CAT model (category approximation of GAMMA model of rate heterogeneity) and the Jones–Taylor–Thornton substitution matrix. Predicted conserved domains were visualized using iTOL. Different conserved domains are represented by colored shapes as indicated in the legend. The evolutionary branches corresponding to Arabidopsis, rice, and wheat are signed with yellow, red, and green circles, respectively.

Additional file 2: Figure S2. Enrichment analysis of conserved motifs at ATP-binding sites (A) and kinase active sites (B). ATP binding sites and kinase active sites were labeled with “*”. Gaps in the sequence alignment are signed by “–”.

Additional file 3: Figure S3. Heatmap of differentially expressed *TaRLKs* in response to various stresses. Differentially expressed genes (DEGs) were identified by filtering out genes with TPM values below 0.5 in the control group. Genes with absolute log_2_FC values greater than 2 under various stress conditions were considered DEGs. A. DEGs induced by heat stress in the wheat variety TAM107 at 1 h and 6 h. B. DEGs induced by drought stress (PEG6000 treatment) in two genotypes Giza 168 (drought tolerant) and Gimmeza 10 (drought sensitive) at 2 h. C. DEGs induced by chitin and flg22 in Chinese Spring variety. D. DEGs induced by *F. pseudograminearum* in two genotypes Janz*2 NIL1 resistant and Janz*2 NIL1 susceptible at 5 d. E. DEGs induced by *F. graminearum* in two genotypes NIL38 (carrying QTL *Fhb1* and *Qfhs.ifa-5A*) and NIL51 (lacking these two QTLs) at 48 h. The heatmap legend represents log_2_FC values, red represents a high expression level and blue represents a low expression level.

Additional file 4: Figure S4. Phylogenetic analysis of the CrRLK1L subfamily genes in Arabidopsis, rice and wheat. The protein sequences of AtCrRLK1Ls (Cheung and Wu 2011), OsCrRLK1Ls (Nguyen et al. 2014) and TaCrRLK1Ls were aligned results using MAFFT with L-INS-I strategy. The phylogenetic tree was constructed using the IQ-TREE 2 with the maximum likelihood method based on the JTT + F + R8 substitution model and supported by 1,000 bootstrap replications.

Additional file 5: Figure S5. Alignment of the amino acid sequences for the three copies of TaCrRLK1L16, HERK and OsCrRLK17. TaCrRLK1L16-4A, TaCrRLK1L16-4B, and TaCrRLK1L16-4D represent TaCrRLK1L16 proteins encoded by CDS nucleotide sequences derived from wheat A, B or D genome, respectively. HERK and OsCrRLK17 are homologous proteins of TaCrRLK1L16 in Arabidopsis and rice, respectively. The protein sequences were aligned with L-INS-I strategy using MAFFT. The signal peptide, malectin domain, transmembrane domain and kinase domain were marked with red underline, respectively.

Additional file 6: Figure S6. Expression patterns of *TaCrRLK1L* family genes in the incompatible group (NIL_R vs. CYR32). The expression patterns of TaCrRLK1L family genes were analyzed in the incompatible group (NIL_R vs. CYR32) using log_2_FC at 0, 18, 24, 48, 96, and 168 hpi, based on time-series dual RNA-seq data. The legend shows the log_2_FC values, where red represents a high expression level and blue represents a low expression level.

Additional file 7: Table S1. Detailed information of 3,424 predicted *TaRLKs*.

Additional file 8: Table S2. Orthologous groups of wheat, rice and Arabidopsis identified by OrthoFinder.

Additional file 9: Table S3. The Ka/Ks ratio values of duplication gene pairs of *TaRLKs*.

Additional file 10: Table S4. TPM values of *TaRLKs* in compatible (NIL_R vs. V26) and incompatible (NIL_R vs. CYR32) interaction between wheat-*Pst*. The transcript levels of *TaRLKs* were analyzed by time series dual RNA-seq data. Two interaction combinations, NIL_R vs. V26 (compatible group) and NIL_R vs. CYR32 (incompatible group), were sequenced with time points at 0, 18, 24, 48, 96 and 168 h post inoculation (hpi). The methodology followed our previous study, and the sequencing data have been submitted to CNSA (https://db.cngb.org/cnsa/) of CNGBdb with accession code CNP0001524 (Bai et al. 2021).

Additional file 11: Table S5. Source entries of *TaRLKs* gene expression data. The expression profiles of wheat variety “Chinese Spring” were obtained from the Wheat Expression Browser powered by expVIP (Borrill et al. 2016). Based on the gene IDs, the expression data of *TaRLKs* were retrieved under various abiotic and biotic stress conditions, including chitin, flg22, heat, PEG6000, *F. graminearum* and *F. pseudograminearum*.

Additional file 12: Table S6. Renamed list of *TaCrRLK1L* subfamily genes.

Additional file 13: Table S7. Primers used in this study.

## Data Availability

All data and materials are available in the paper and online supplemental files.

## Abbreviations

CrRLK1L: *Catharanthus roseus* Receptor-like kinase 1-like
ECDs: Extracellular domains
ETI: Effector-Triggered Immunity
HR: Hypersensitive response
Ka/Ks: Nonsynonymous (Ka) and synonymous (Ks) ratio
ORF: Open reading frame
PRRs: Pattern Recognition Receptors
Pst: *Puccinia striiformis* f. sp. *tritici*
PTI: Pattern-Triggered Immunity
RALF: Rapid Alkalinization Factor
RLCKs: Receptor-Like Cytoplasmic Kinases
RLKs: Receptor-like kinases
RTKs: Receptor Tyrosine Kinases
TaR LKs: Wheat (*Triticum aestivum*) Receptor-like kinases
vWA: Von Willebrand factor A
WAK: Wall-associated receptor kinase

## References

[CR1] Almagro Armenteros JJ, Tsirigos KD, Sønderby CK, Petersen TN, Winther O, Brunak S, von Heijne G, Nielsen H (2019) SignalP 5.0 improves signal peptide predictions using deep neural networks. Nat Biotechnol 37:420–423. 10.1038/s41587-019-0036-z 30778233 10.1038/s41587-019-0036-z

[CR2] Ausubel FM (2005) Are innate immune signaling pathways in plants and animals conserved? Nat Immunol 6:973–979. 10.1038/ni1253 16177805 10.1038/ni1253

[CR3] Bai X, Zhan G, Tian S, Peng H, Cui X, Islam MA, Goher F, Ma Y, Kang Z, Xu ZS, Guo J (2021) Transcription factor BZR2 activates chitinase *Cht20.2* transcription to confer resistance to wheat stripe rust. Plant Physiol 187:2749–2762. 10.1093/plphys/kiab383 34618056 10.1093/plphys/kiab383 PMC8644182

[CR4] Borrill P, Ramirez-Gonzalez R, Uauy C (2016) expVIP: A customizable RNA-seq data analysis and visualization platform. Plant Physiol 170:2172–2186. 10.1104/pp.15.01667 26869702 10.1104/pp.15.01667 PMC4825114

[CR5] Chen C, Wu Y, Li J, Wang X, Zeng Z, Xu J, Liu Y, Feng J, Chen H, He Y, Xia R (2023) TBtools-II: A “one for all, all for one” bioinformatics platform for biological big-data mining. Mol Plant 16:1733–1742. 10.1016/j.molp.2023.09.010 37740491 10.1016/j.molp.2023.09.010

[CR6] Cheung AY, Wu H-M (2011) THESEUS 1, FERONIA and relatives: a family of cell wall-sensing receptor kinases? Curr Opin Plant Biol 14:632–641. 10.1016/j.pbi.2011.09.001 21963060 10.1016/j.pbi.2011.09.001

[CR7] Chisholm ST, Coaker G, Day B, Staskawicz BJ (2006) Host-microbe interactions: shaping the evolution of the plant immune response. Cell 124:803–814. 10.1016/j.cell.2006.02.008 16497589 10.1016/j.cell.2006.02.008

[CR8] Dangl JL, Jones JDG (2001) Plant pathogens and integrated defence responses to infection. Nature 411:826–833. 10.1038/35081161 11459065 10.1038/35081161

[CR9] Dievart A, Gottin C, Périn C, Ranwez V, Chantret N (2020) Origin and diversity of plant receptor-like kinases. Annu Rev Plant Biol 71:131–156. 10.1146/annurev-arplant-073019-025927 32186895 10.1146/annurev-arplant-073019-025927

[CR10] Dou D, Zhou J-M (2012) Phytopathogen effectors subverting host immunity: different foes, similar battleground. Cell Host Microbe 12:484–495. 10.1016/j.chom.2012.09.003 23084917 10.1016/j.chom.2012.09.003

[CR11] Emms DM, Kelly S (2019) OrthoFinder: phylogenetic orthology inference for comparative genomics. Genome Biol 20:238. 10.1186/s13059-019-1832-y 31727128 10.1186/s13059-019-1832-y PMC6857279

[CR12] Fan J, Bai P, Ning Y, Wang J, Shi X, Xiong Y , Zhang K, He F, Zhang C, Wang R, Meng X, Zhou J, Wang M, Shirsekar G, Park CH, Bellizzi M, Liu W, Jeon JS, Xia Y, Shan L, Wang GL (2018) The monocot-specific receptor-like kinase SDS2 controls cell death and immunity in rice. Cell Host Microbe 23:498-510.e5. 10.1016/j.chom.2018.03.003 29576481 10.1016/j.chom.2018.03.003 PMC6267930

[CR13] Gan P, Tang C, Lu Y, Ren C, Nasab HR, Kun X, Wang X, Li L, Kang Z, Wang X, Wang J (2024) Quantitative phosphoproteomics reveals molecular pathway network in wheat resistance to stripe rust. Stress Biol 4:32. 10.1007/s44154-024-00170-0 38945963 10.1007/s44154-024-00170-0 PMC11214938

[CR14] Gao C-H, Yu G, Cai P (2021) ggVennDiagram: An intuitive, easy-to-use, and highly customizable r package to generate venn diagram. Front Genet 12:706907. 10.3389/fgene.2021.706907 34557218 10.3389/fgene.2021.706907 PMC8452859

[CR15] Gasteiger E, Hoogland C, Gattiker A, Duvaud S, Wilkins MR, Appel RD, Bairoch A (2005) Protein identification and analysis tools on the ExPASy server. In: Walker JM (ed) The Proteomics Protocols Handbook. Humana Press, Totowa, pp 571–607. 10.1385/1-59259-890-0:571

[CR16] Gómez-Gómez L, Boller T (2000) FLS2: An LRR receptor–like kinase involved in the perception of the bacterial elicitor flagellin in Arabidopsis. Mol Cell 5:1003–1011. 10.1016/S1097-2765(00)80265-8 10911994 10.1016/s1097-2765(00)80265-8

[CR17] Gu J, Sun J, Liu N, Sun X, Liu C, Wu L, Liu G, Zeng F, Hou C, Han S, Zhen W, Wang D (2020) A novel cysteine-rich receptor-like kinase gene, *TaCRK2*, contributes to leaf rust resistance in wheat. Mol Plant Pathol 21:732–746. 10.1111/mpp.12929 32196909 10.1111/mpp.12929 PMC7170779

[CR18] Guo J, Islam MA, Lin H, Ji C, Duan Y, Liu P, Zeng Q, Day B, Kang Z, Guo J (2018) Genome-wide identification of cyclic nucleotide-gated ion channel gene family in wheat and functional analyses of *TaCNGC14* and *TaCNGC16*. Front Plant Sci 9:18. 10.3389/fpls.2018.00018 10.3389/fpls.2018.00018 PMC5786745 29403523

[CR19] Hayta S, Smedley MA, Demir SU, Blundell R, Hinchliffe A, Atkinson N, Harwood WA (2019) An efficient and reproducible Agrobacterium-mediated transformation method for hexaploid wheat (*Triticum aestivum* L.). Plant Methods 15:121. 10.1186/s13007-019-0503-z 31673278 10.1186/s13007-019-0503-z PMC6815027

[CR20] Hooper CM, Castleden IR, Aryamanesh N, Jacoby RP, Millar AH (2015) Finding the subcellular location of barley, wheat, rice and maize proteins: the compendium of crop proteins with annotated locations (cropPAL). Plant Cell Physiol 57:e9–e9. 10.1093/pcp/pcv170 26556651 10.1093/pcp/pcv170

[CR21] Huang X, Liu Y, Jia Y, Ji L, Luo X, Tian S, Chen T (2024) FERONIA homologs in stress responses of horticultural plants: current knowledge and missing links. Stress Biol 4:28. 10.1007/s44154-024-00161-1 38847988 10.1007/s44154-024-00161-1 PMC11161445

[CR22] Jones JDG, Dangl JL (2006) The plant immune system. Nature 444:323–329. 10.1038/nature05286 17108957 10.1038/nature05286

[CR23] Koike M, Sutoh K, Kawakami A, Torada A, Oono K, Imai R (2005) Molecular characterization of a cold-induced plasma membrane protein gene from wheat. Mol Genet Genomics 274:445–453. 10.1007/s00438-005-0050-3 16184390 10.1007/s00438-005-0050-3

[CR24] Kadota Y, Sklenar J, Derbyshire P, Stransfeld L, Asai S, Ntoukakis V, Jones JD, Shirasu K, Menke F, Jones A, Zipfel C (2014) Direct regulation of the NADPH oxidase RBOHD by the PRR-associated kinase BIK1 during plant immunity. Mol Cell 54:43–55. 10.1016/j.molcel.2014.02.021 24630626 10.1016/j.molcel.2014.02.021

[CR25] Katoh K, Standley DM (2013) MAFFT multiple sequence alignment software version 7: improvements in performance and usability. Mol Biol Evol 30:772–780. 10.1093/molbev/mst010 23329690 10.1093/molbev/mst010 PMC3603318

[CR26] Krogh A, Larsson B, von Heijne G, Sonnhammer ELL (2001) Predicting transmembrane protein topology with a hidden markov model: application to complete genomes. J Mol Biol 305:567–580. 10.1006/jmbi.2000.4315 11152613 10.1006/jmbi.2000.4315

[CR27] Letunic I, Bork P (2021) Interactive tree of life (iTOL) v5: An online tool for phylogenetic tree display and annotation. Nucleic Acids Res 49:W293–W296. 10.1093/nar/gkab301 33885785 10.1093/nar/gkab301 PMC8265157

[CR28] Letunic I, Khedkar S, Bork P (2020) SMART: recent updates, new developments and status in 2020. Nucleic Acids Res 49:D458–D460. 10.1093/nar/gkaa937 10.1093/nar/gkaa937 PMC7778883 33104802

[CR29] Liang X, Zhang J (2022) Regulation of plant responses to biotic and abiotic stress by receptor-like cytoplasmic kinases. Stress Biol 2:25. 10.1007/s44154-022-00045-2 37676353 10.1007/s44154-022-00045-2 PMC10441961

[CR30] Liang X, Zhou J-M (2018) Receptor-Like Cytoplasmic Kinases: Central players in plant receptor kinase-mediated signaling. Annu Rev Plant Biol 69:267–299. 10.1146/annurev-arplant-042817-040540 29719165 10.1146/annurev-arplant-042817-040540

[CR31] Liu MJ, Yeh FJ, Yvon R, Simpson K, Jordan S, Chambers J, Wu HM, Cheung AY (2024a) Extracellular pectin-RALF phase separation mediates FERONIA global signaling function. Cell 187:312-330.e22. 10.1016/j.cell.2023.11.038 38157854 10.1016/j.cell.2023.11.038

[CR32] Liu Q, Fu Q, Yan Y, Jiang Q, Mao L, Wang L, Yu F, Zheng H (2024b) Curation, nomenclature, and topological classification of receptor-like kinases from 528 plant species for novel domain discovery and functional inference. Mol Plant 17:658–671. 10.1016/j.molp.2024.02.015 38384130 10.1016/j.molp.2024.02.015

[CR33] Lu S, Wang J, Chitsaz F, Derbyshire MK, Geer RC, Gonzales NR, Gwadz M, Hurwitz DI, Marchler GH, Song JS, Thanki N, Yamashita RA, Yang M, Zhang D, Zheng C, Lanczycki CJ, Marchler-Bauer A (2019) CDD/SPARCLE: The conserved domain database in 2020. Nucleic Acids Res 48:D265–D268. 10.1093/nar/gkz991 10.1093/nar/gkz991 PMC6943070 31777944

[CR34] Macho A, Wang P, Zhu J-K (2022) Modification of the susceptibility gene TaPsIPK1 - a win-win for wheat disease resistance and yield. Stress Biol 2:40. 10.1007/s44154-022-00060-3 37676463 10.1007/s44154-022-00060-3 PMC10441897

[CR35] Minh BQ, Schmidt HA, Chernomor O, Schrempf D, Woodhams MD, von Haeseler A, Lanfear R (2020) IQ-TREE 2: new models and efficient methods for phylogenetic inference in the genomic era. Mol Biol Evol 37:1530–1534. 10.1093/molbev/msaa015 32011700 10.1093/molbev/msaa015 PMC7182206

[CR36] Mistry J, Chuguransky S, Williams L, Qureshi M, Salazar GA, Sonnhammer ELL, Tosatto SCE, Paladin L, Raj S, Richardson LJ, Finn RD, Bateman A (2020) Pfam: The protein families database in 2021. Nucleic Acids Res 49:D412–D419. 10.1093/nar/gkaa913 10.1093/nar/gkaa913 PMC7779014 33125078

[CR37] Ngou BPM, Ding P, Jones JDG (2022) Thirty years of resistance: Zig-zag through the plant immune system. Plant Cell 34:1447–1478. 10.1093/plcell/koac041 35167697 10.1093/plcell/koac041 PMC9048904

[CR38] Nguyen QN, Lee YS, Cho LH, Jeong HJ, An G, Jung KH (2014) Genome-wide identification and analysis of *Catharanthus roseus* RLK1-like kinases in rice. Planta 241:603–613. 10.1007/s00425-014-2203-2 25399351 10.1007/s00425-014-2203-2

[CR39] Petutschnig EK, Jones AME, Serazetdinova L, Lipka U, Lipka V (2010) The lysin motif receptor-like kinase (LysM-RLK) CERK1 is a major chitin-binding protein in *Arabidopsis thaliana* and subject to Chitin-induced phosphorylation. J Biol Chem 285:28902–28911. 10.1074/jbc.M110.116657 20610395 10.1074/jbc.M110.116657 PMC2937917

[CR40] Pitsili E, Phukan UJ, Coll NS (2020) Cell death in plant immunity. Cold Spring Harb Perspect Biol 12:a036483. 10.1101/cshperspect.a036483 31615784 10.1101/cshperspect.a036483 PMC7263082

[CR41] Price MN, Dehal PS, Arkin AP (2010) FastTree 2 – approximately maximum-likelihood trees for large alignments. PLoS ONE 5:e9490. 10.1371/journal.pone.0009490 20224823 10.1371/journal.pone.0009490 PMC2835736

[CR42] Raivo K (2010) pheatmap: Pretty Heatmaps. 1.0.12. 10.32614/CRAN.package.pheatmap

[CR43] Ramírez-González RH, Borrill P, Lang D, Harrington HA, Brinton J, Venturini L, Davey M, Jacobs J, F van Ex, Pasha A et al (2018) The transcriptional landscape of polyploid wheat. Science 361:eaar6089. 10.1126/science.aar6089 30115782 10.1126/science.aar6089

[CR44] Rao S, Zhou Z, Miao P, Bi G, Hu M, Wu Y, Feng F, Zhang X, Zhou JM (2018) Roles of receptor-like cytoplasmic kinase VII members in pattern-triggered immune signaling. Plant Physiol pp.00486.2018. 10.1104/pp.18.00486 10.1104/pp.18.00486 PMC6084675 29907700

[CR45] Saintenac C, Cambon F, Aouini L, Verstappen E, Ghaffary SMT, Poucet T, Marande W, Berges H, Xu S, Jaouannet M, Favery B, Alassimone J, Sánchez-Vallet A, Faris J, Kema G, Robert O, Langin T (2021) A wheat cysteine-rich receptor-like kinase confers broad-spectrum resistance against Septoria tritici blotch. Nat Commun 12:433. 10.1038/s41467-020-20685-0 33469010 10.1038/s41467-020-20685-0 PMC7815785

[CR46] Schlessinger J (2014) Receptor tyrosine kinases: legacy of the first two decades. Cold Spring Harb Perspect Biol 6:a008912–a008912. 10.1101/cshperspect.a008912 24591517 10.1101/cshperspect.a008912 PMC3949355

[CR47] Shi Y, Bao X, Song X, Liu Y, Li Y, Chen X, Hu X (2023) The leucine-rich repeat receptor-like kinase protein TaSERK1 positively regulates high-temperature seedling plant resistance to *Puccinia striiformis* f. sp. *tritici* by interacting with TaDJA7. Phytopathology 113:1325–1334. 10.1094/PHYTO-11-22-0429-R 36774558 10.1094/PHYTO-11-22-0429-R

[CR48] Shiu S-H, Bleecker AB (2001) Plant receptor-like kinase gene family: diversity, function, and signaling. Sci STKE 2001:re22. 10.1126/stke.2001.113.re22 11752632 10.1126/stke.2001.113.re22

[CR49] Shiu SH, Karlowski WM, Pan R, Tzeng YH, Mayer KF, Li WH (2004) Comparative analysis of the receptor-like kinase family in Arabidopsis and Rice. Plant Cell 16:1220–1234. 10.1105/tpc.020834 15105442 10.1105/tpc.020834 PMC423211

[CR50] Teixeira PJP, Colaianni NR, Fitzpatrick CR, Dangl JL (2019) Beyond pathogens: microbiota interactions with the plant immune system. Curr Opin Microbiol 49:7–17. 10.1016/j.mib.2019.08.003 31563068 10.1016/j.mib.2019.08.003

[CR51] Tian W, Hou C, Ren Z, Wang C, Zhao F, Dahlbeck D, Hu S, Zhang L, Niu Q, Li L, Staskawicz BJ, Luan S (2019) A calmodulin-gated calcium channel links pathogen patterns to plant immunity. Nature 572:131–135. 10.1038/s41586-019-1413-y 31316205 10.1038/s41586-019-1413-y

[CR52] Tian H, Wu Z, Chen S, Ao K, Huang W, Yaghmaiean H, Sun T, Xu F, Zhang Y, Wang S, Li X, Zhang Y (2021) Activation of TIR signalling boosts pattern-triggered immunity. Nature 598:500–503. 10.1038/s41586-021-03987-1 34544113 10.1038/s41586-021-03987-1

[CR53] Walker JC, Zhang R (1990) Relationship of a putative receptor protein kinase from maize to the S-locus glycoproteins of Brassica. Nature 345:743–746. 10.1038/345743a0 2163028 10.1038/345743a0

[CR54] Wang C-F, Huang L-L, Buchenauer H, Han Q-M, Zhang H-C, Kang Z-S (2007) Histochemical studies on the accumulation of reactive oxygen species (O_2_^−^ and H_2_O_2_) in the incompatible and compatible interaction of wheat-*Puccinia striiformis* f. sp. *tritici*. Physiol Mol Plant Pathol 71:230–239. 10.1016/j.pmpp.2008.02.006

[CR55] Wang J, Wang J, Shang H, Chen X, Xu X, Hu X (2019) *TaXa21*, a leucine-rich repeat receptor-like kinase gene associated with *TaWRKY76* and *TaWRKY62*, plays positive roles in wheat high-temperature seedling plant resistance to *Puccinia striiformis* f. sp. *tritici*. Mol Plant-Microbe Interactions 32:1526–1535. 10.1094/MPMI-05-19-0137-R 10.1094/MPMI-05-19-0137-R 31237476

[CR56] Wang N, Tang C, Fan X, He M, Gan P, Zhang S, Hu Z, Wang X, Yan T, Shu W et al (2022) Inactivation of a wheat protein kinase gene confers broad-spectrum resistance to rust fungi. Cell 185:2961-2974.e19. 10.1016/j.cell.2022.06.027 35839760 10.1016/j.cell.2022.06.027

[CR57] Wang Y, Abrouk M, Gourdoupis S, Koo DH, Karafiátová M, Molnár I, Holušová K, Doležel J, Athiyannan N, Cavalet-Giorsa E, Jaremko Ł, Poland J, Krattinger SG (2023) An unusual tandem kinase fusion protein confers leaf rust resistance in wheat. Nat Genet 55:914–920. 10.1038/s41588-023-01401-2 37217716 10.1038/s41588-023-01401-2 PMC10260399

[CR58] Wang Y, Liu X, Yuan B, Chen X, Zhao H, Ali Q, Zheng M, Tan Z, Yao H, Zheng S, Wu J, Xu J, Shi J, Wu H, Gao X, Gu Q (2024) *Fusarium graminearum* rapid alkalinization factor peptide negatively regulates plant immunity and cell growth via the FERONIA receptor kinase. Plant Biotechnol J 22:1800–1811. 10.1111/pbi.14303 10.1111/pbi.14303 PMC11182587 38344883

[CR59] Wu Z, Zhang G, Zhao R, Gao Q, Zhao J, Zhu X, Wang F, Kang Z, Wang X (2023) Transcriptomic analysis of wheat reveals possible resistance mechanism mediated by *Yr10* to stripe rust. Stress Biol 3:44. 10.1007/s44154-023-00115-z 10.1007/s44154-023-00115-z PMC10593697 37870601

[CR60] Xie J, Chen Y, Cai G, Cai R, Hu Z, Wang H (2023) Tree Visualization By One Table (tvBOT): a web application for visualizing, modifying and annotating phylogenetic trees. Nucleic Acids Res 51:W587–W592. 10.1093/nar/gkad359 37144476 10.1093/nar/gkad359 PMC10320113

[CR61] Xu L, Wang J, Xiao Y, Han Z, Chai J (2023) Structural insight into chitin perception by chitin elicitor receptor kinase 1 of *Oryza sativa*. J Integr Plant Biol 65:235–248. 10.1111/jipb.13279 10.1111/jipb.13279 35568972

[CR62] Yadeta KA, Elmore JM, Creer AY, Feng B, Franco JY, Rufian JS, He P, Phinney B, Coaker G (2017) A cysteine-rich protein kinase associates with a membrane immune complex and the cysteine residues are required for cell death. Plant Physiol 173:771–787. 10.1104/pp.16.01404 27852951 10.1104/pp.16.01404 PMC5210739

[CR63] Yin Z, Liu J, Dou D (2024) RLKdb: A comprehensively curated database of plant receptor-like kinase families. Mol Plant 17:513–515. 10.1016/j.molp.2024.02.014 38384129 10.1016/j.molp.2024.02.014

[CR64] Zhang L, Wang J-C, Hou L, Cao PR, Wu L, Zhang QS, Yang HY, Zang Y, Ding JP, Li J (2015) Functional role of histidine in the conserved His-x-Asp motif in the catalytic core of protein kinases. Sci Rep 5:10115. 10.1038/srep10115 25960268 10.1038/srep10115 PMC4650784

[CR65] Zhang R, Shi P-T, Zhou M, Liu HZ, Xu XJ, Liu WT, Chen KM (2023) Rapid alkalinization factor: function, regulation, and potential applications in agriculture. Stress Biol 3:16. 10.1007/s44154-023-00093-2 37676530 10.1007/s44154-023-00093-2 PMC10442051

[CR66] Zhang L, Zhu Q, Tan Y, Deng M, Zhang L, Cao Y, Guo X (2024) Mitogen-activated protein kinases MPK3 and MPK6 phosphorylate receptor-like cytoplasmic kinase CDL1 to regulate soybean basal immunity. Plant Cell 36:963–986. 10.1093/plcell/koae008 38301274 10.1093/plcell/koae008 PMC10980351

[CR67] Zhao J, Kang Z (2023) Fighting wheat rusts in China: a look back and into the future. Phytopathol Res 5:6. 10.1186/s42483-023-00159-z

[CR68] Zhou H, Li S, Deng Z, Wang X, Chen T, Zhang J, Chen S, Ling H, Zhang A, Wang D, Zhang X (2007) Molecular analysis of three new receptor-like kinase genes from hexaploid wheat and evidence for their participation in the wheat hypersensitive response to stripe rust fungus infection. Plant J 52:420–434. 10.1111/j.1365-313X.2007.03246.x 17764502 10.1111/j.1365-313X.2007.03246.x

[CR69] Zhu S, Fu Q, Xu F, Zheng H, Yu F (2021) New paradigms in cell adaptation: decades of discoveries on the *Cr*RLK1L receptor kinase signalling network. New Phytol 232:1168–1183. 10.1111/nph.17683 34424552 10.1111/nph.17683

[CR70] Zipfel C (2014) Plant pattern-recognition receptors. Trends Immunol 35:345–351. 10.1016/j.it.2014.05.004 24946686 10.1016/j.it.2014.05.004

[CR71] Zipfel C, Kunze G, Chinchilla D, Caniard A, Jones JD, Boller T, Felix G (2006) Perception of the Bacterial PAMP EF-Tu by the Receptor EFR Restricts Agrobacterium-Mediated Transformation. Cell 125:749–760. 10.1016/j.cell.2006.03.037 16713565 10.1016/j.cell.2006.03.037

