# Supplementary material for: Evolutionary characteristics, expression patterns of wheat receptor-like kinases and functional analysis of TaCrRLK1L16
Source: Stress Biol. 2025 Apr 3;5(1):24. doi: 10.1007/s44154-025-00215-y (PMC11968617; doi:10.1007/s44154-025-00215-y)
Supplement: Supplementary file 1 — Additional file 1: Figure S1. Phylogenetic analysis of RLKs in wheat, rice and Arabidopsis and 3,424 conserved domains of TaRLKs. The protein sequences of 3,424 TaRLKs, 613 AtRLKs and 1,087 OsRLKs were aligned using MAFFT with the L-INS-I strategy. The maximum-likelihood tree was constructed with Fasttree using the CAT model (category approximation of GAMMA model of rate heterogeneity) and the Jones–Taylor–Thornton substitution matrix. Predicted conserved domains were visualized using iTOL. Different conserved domains are represented by colored shapes as indicated in the legend. The evolutionary branches corresponding to Arabidopsis, rice, and wheat are signed with yellow, red, and green circles, respectively. [file 44154_2025_215_MOESM1_ESM.pdf]

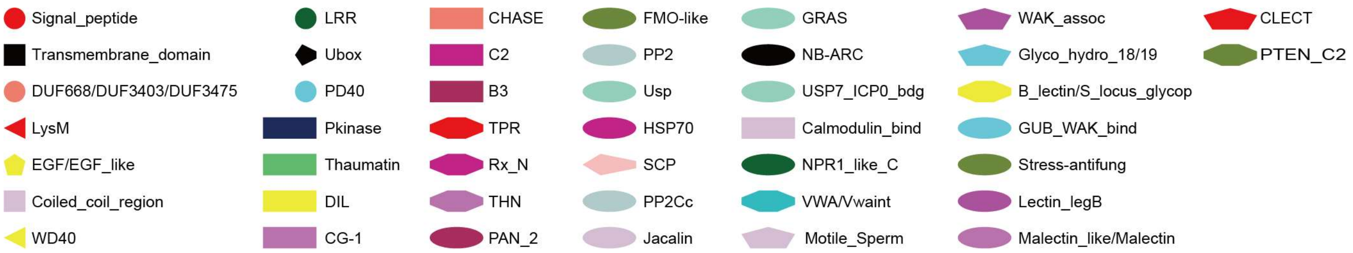

Figure S1. Phylogenetic analysis of RLKs in wheat, rice and Arabidopsis and 3,424 conserved domains of TaRLKs. The protein sequences of 3,424 TaRLKs, 613 AtRLKs and 1,087 OsRLKs were aligned using MAFFT with the L-INS-I strategy. The maximum-likelihood tree was constructed with Fasttree using the CAT model (category approximation of GAMMA model of rate heterogeneity) and the Jones–Taylor–Thornton substitution matrix. Predicted conserved domains were visualized using iTOL. Different conserved domains are represented by colored shapes as indicated in the legend. The evolutionary branches corresponding to Arabidopsis, rice, and wheat are signed with yellow, red, and green circles, respectively.
