# Supplementary material for: Evolutionary characteristics, expression patterns of wheat receptor-like kinases and functional analysis of TaCrRLK1L16
Source: Stress Biol. 2025 Apr 3;5(1):24. doi: 10.1007/s44154-025-00215-y (PMC11968617; doi:10.1007/s44154-025-00215-y)
Supplement: Supplementary file 2 — Additional file 2: Figure S2. Enrichment analysis of conserved motifs at ATP-binding sites (A) and kinase active sites (B). ATP binding sites and kinase active sites were labeled with “*”. Gaps in the sequence alignment are signed by “–”. [file 44154_2025_215_MOESM2_ESM.pdf]

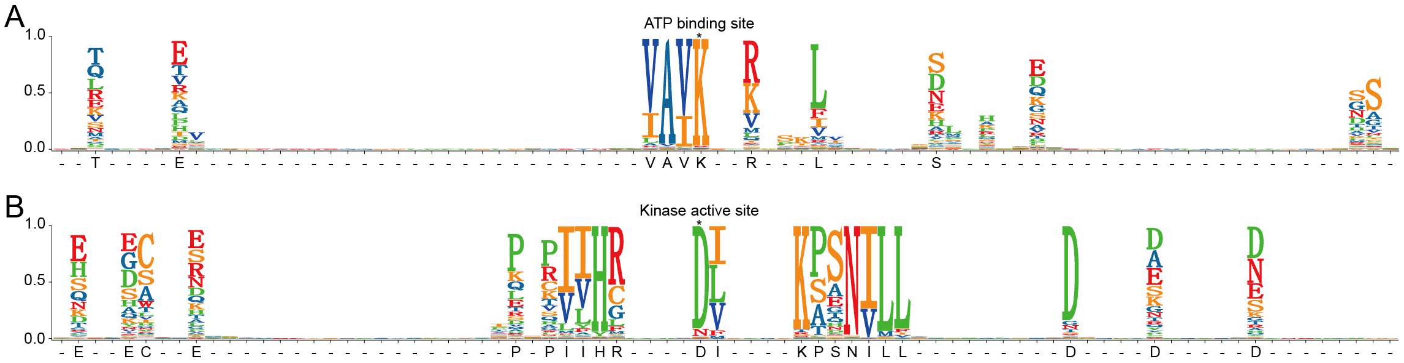

Figure S2. Enrichment analysis of conserved motifs at ATP-binding sites (**A**) and kinase active sites (**B**). ATP binding sites and kinase active sites were labeled with "*". Gaps in the sequence alignment are signed by "–".
