# Supplementary material for: Evolutionary characteristics, expression patterns of wheat receptor-like kinases and functional analysis of TaCrRLK1L16
Source: Stress Biol. 2025 Apr 3;5(1):24. doi: 10.1007/s44154-025-00215-y (PMC11968617; doi:10.1007/s44154-025-00215-y)
Supplement: Supplementary file 3 — Additional file 3: Figure S3. Heatmap of differentially expressed TaRLKs in response to various stresses. Differentially expressed genes (DEGs) were identified by filtering out genes with TPM values below 0.5 in the control group. Genes with absolute log2FC values greater than 2 under various stress conditions were considered DEGs. A. DEGs induced by heat stress in the wheat variety TAM107 at 1 h and 6 h. B. DEGs induced by drought stress (PEG6000 treatment) in two genotypes Giza 168 (drought tolerant) and Gimmeza 10 (drought sensitive) at 2 h. C. DEGs induced by chitin and flg22 in Chinese Spring variety. D. DEGs induced by F. pseudograminearum in two genotypes Janz*2 NIL1 resistant and Janz*2 NIL1 susceptible at 5 d. E. DEGs induced by F. graminearum in two genotypes NIL38 (carrying QTL Fhb1 and Qfhs.ifa-5A) and NIL51 (lacking these two QTLs) at 48 h. The heatmap legend represents log2FC values, red represents a high expression level and blue represents a low expression level. [file 44154_2025_215_MOESM3_ESM.pdf]

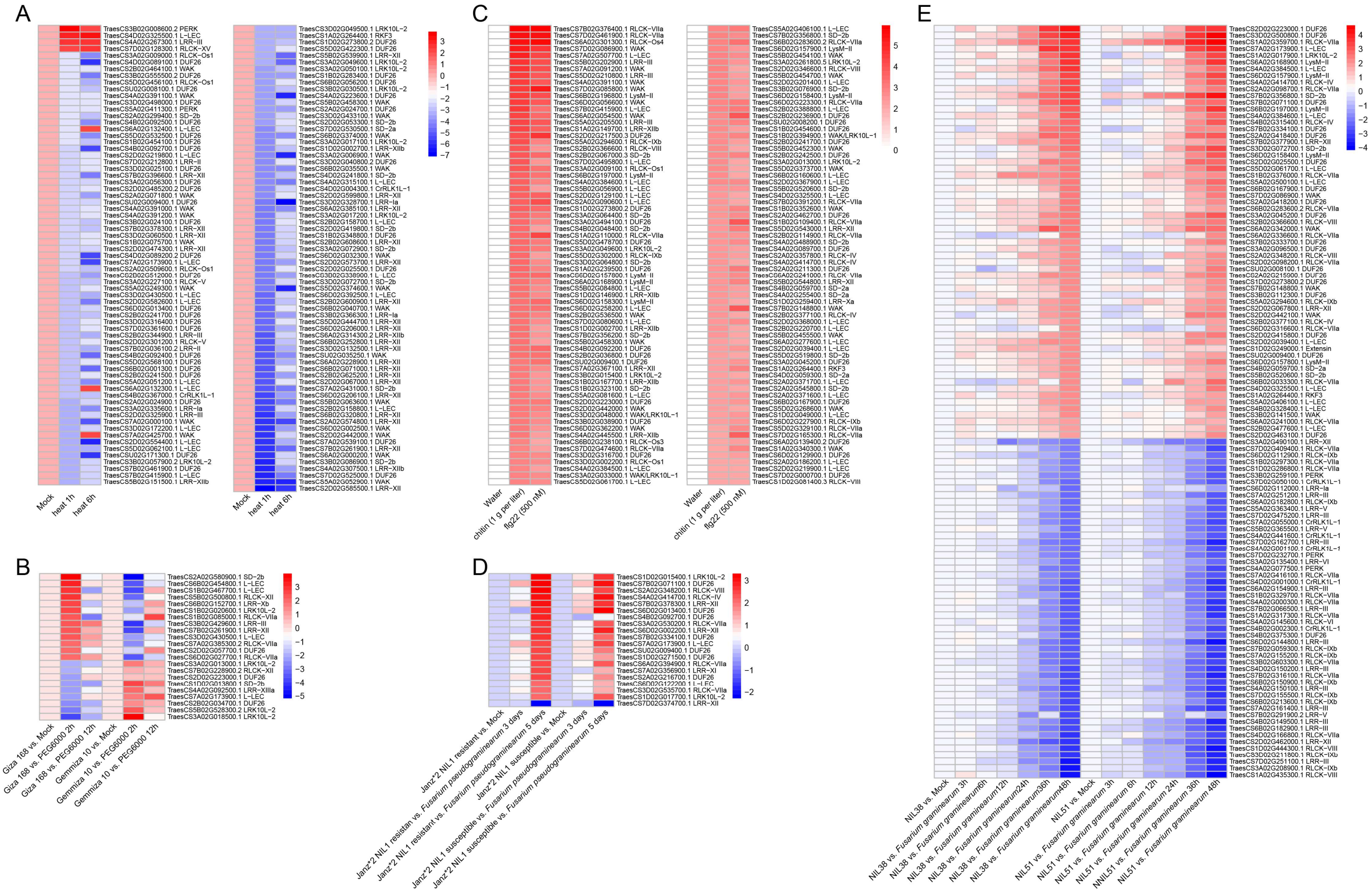

Figure S3. Heatmap of differentially expressed *TaRLKs* in response to various stresses. Differentially expressed genes (DEGs) were identified by filtering out genes with TPM values below 0.5 in the control group. Genes with absolute log_2FC values greater than 2 under various stress conditions were considered DEGs. A. DEGs induced by heat stress in the wheat variety TAM107 at 1 h and 6 h. B. DEGs induced by drought stress (PEG6000 treatment) in two genotypes Giza 168 (drought tolerant) and Gimmeza 10 (drought sensitive) at 2 h. C. DEGs induced by chitin and flg22 in Chinese Spring variety. D. DEGs induced by *F. pseudograminearum* in two genotypes Janz*2 NIL1 resistant and Janz*2 NIL1 susceptible at 5 d. E. DEGs induced by *F. graminearum* in two genotypes NIL38 (carrying QTL *Fhb1* and *Qfhs.ifa-5A*) and NIL51 (lacking these two QTLs) at 48 h. The heatmap legend represents log_2FC values, red represents a high expression level and blue represents a low expression level.
