# Supplementary material for: Evolutionary characteristics, expression patterns of wheat receptor-like kinases and functional analysis of TaCrRLK1L16
Source: Stress Biol. 2025 Apr 3;5(1):24. doi: 10.1007/s44154-025-00215-y (PMC11968617; doi:10.1007/s44154-025-00215-y)
Supplement: Supplementary file 5 — Additional file 5: Figure S5. Alignment of the amino acid sequences for the three copies of TaCrRLK1L16, HERK and OsCrRLK17. TaCrRLK1L16-4A, TaCrRLK1L16-4B, and TaCrRLK1L16-4D represent TaCrRLK1L16 proteins encoded by CDS nucleotide sequences derived from wheat A, B or D genome, respectively. HERK and OsCrRLK17 are homologous proteins of TaCrRLK1L16 in Arabidopsis and rice, respectively. The protein sequences were aligned with L-INS-I strategy using MAFFT. The signal peptide, malectin domain, transmembrane domain and kinase domain were marked with red underline, respectively. [file 44154_2025_215_MOESM5_ESM.pdf]

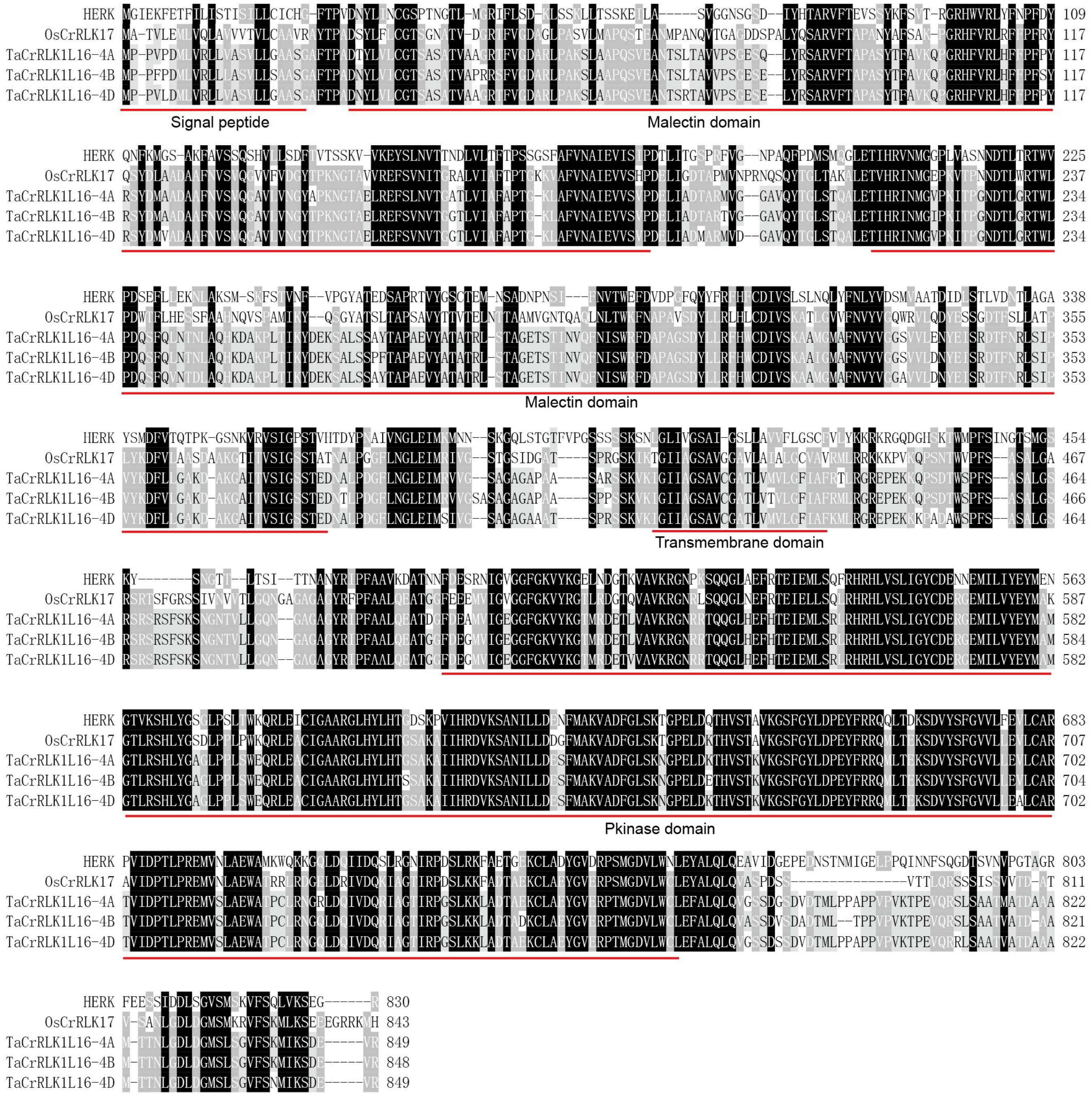

Figure S5. Alignment of the amino acid sequences for the three copies of TaCrRLK1L16, HERK and OsCrRLK17. TaCrRLK1L16-4A, TaCrRLK1L16-4B, and TaCrRLK1L16-4D represent TaCrRLK1L16 proteins encoded by CDS nucleotide sequences derived from wheat A, B or D genome, respectively. HERK and OsCrRLK17 are homologous proteins of TaCrRLK1L16 in Arabidopsis and rice, respectively. The protein sequences were aligned with L-INS-I strategy using MAFFT. The signal peptide, malectin domain, transmembrane domain and kinase domain were marked with red underline, respectively.
