# Supplementary material for: Evolutionary characteristics, expression patterns of wheat receptor-like kinases and functional analysis of TaCrRLK1L16
Source: Stress Biol. 2025 Apr 3;5(1):24. doi: 10.1007/s44154-025-00215-y (PMC11968617; doi:10.1007/s44154-025-00215-y)
Supplement: Supplementary file 6 — Additional file 6: Figure S6. Expression patterns of TaCrRLK1L family genes in the incompatible group (NIL_R vs. CYR32). The expression patterns of TaCrRLK1L family genes were analyzed in the incompatible group (NIL_R vs. CYR32) using log2FC at 0, 18, 24, 48, 96, and 168 hpi, based on time-series dual RNA-seq data. The legend shows the log2FC values, where red represents a high expression level and blue represents a low expression level. [file 44154_2025_215_MOESM6_ESM.pdf]

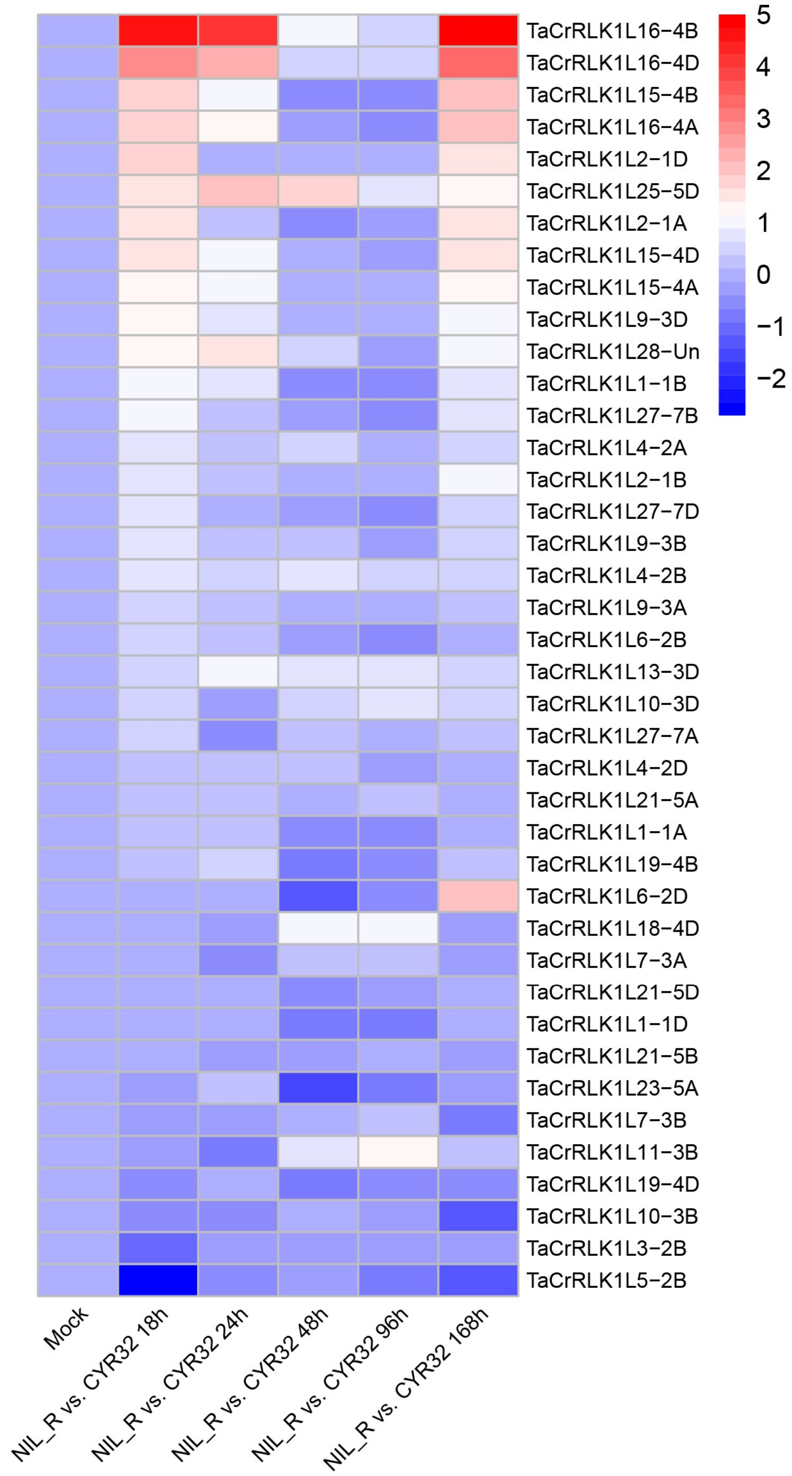

Figure S6. Expression patterns of *TaCrRLK1L* family genes in the incompatible group (NIL_R vs. CYR32). The expression patterns of TaCrRLK1L family genes were analyzed in the incompatible group (NIL_R vs. CYR32) using log_2FC at 0, 18, 24, 48, 96, and 168 hpi, based on time-series dual RNA-seq data. The legend shows the log_2FC values, where red represents a high expression level and blue represents a low expression level.
