# Supplementary material for: Evolutionary characteristics, expression patterns of wheat receptor-like kinases and functional analysis of TaCrRLK1L16
Source: Stress Biol. 2025 Apr 3;5(1):24. doi: 10.1007/s44154-025-00215-y (PMC11968617; doi:10.1007/s44154-025-00215-y)
Supplement: Supplementary file 4 — Additional file 4: Figure S4. Phylogenetic analysis of the CrRLK1L subfamily genes in Arabidopsis, rice and wheat. The protein sequences of AtCrRLK1Ls (Cheung and Wu 2011), OsCrRLK1Ls (Nguyen et al. 2014) and TaCrRLK1Ls were aligned results using MAFFT with L-INS-I strategy. The phylogenetic tree was constructed using the IQ-TREE 2 with the maximum likelihood method based on the JTT + F + R8 substitution model and supported by 1,000 bootstrap replications. [file 44154_2025_215_MOESM4_ESM.pdf]

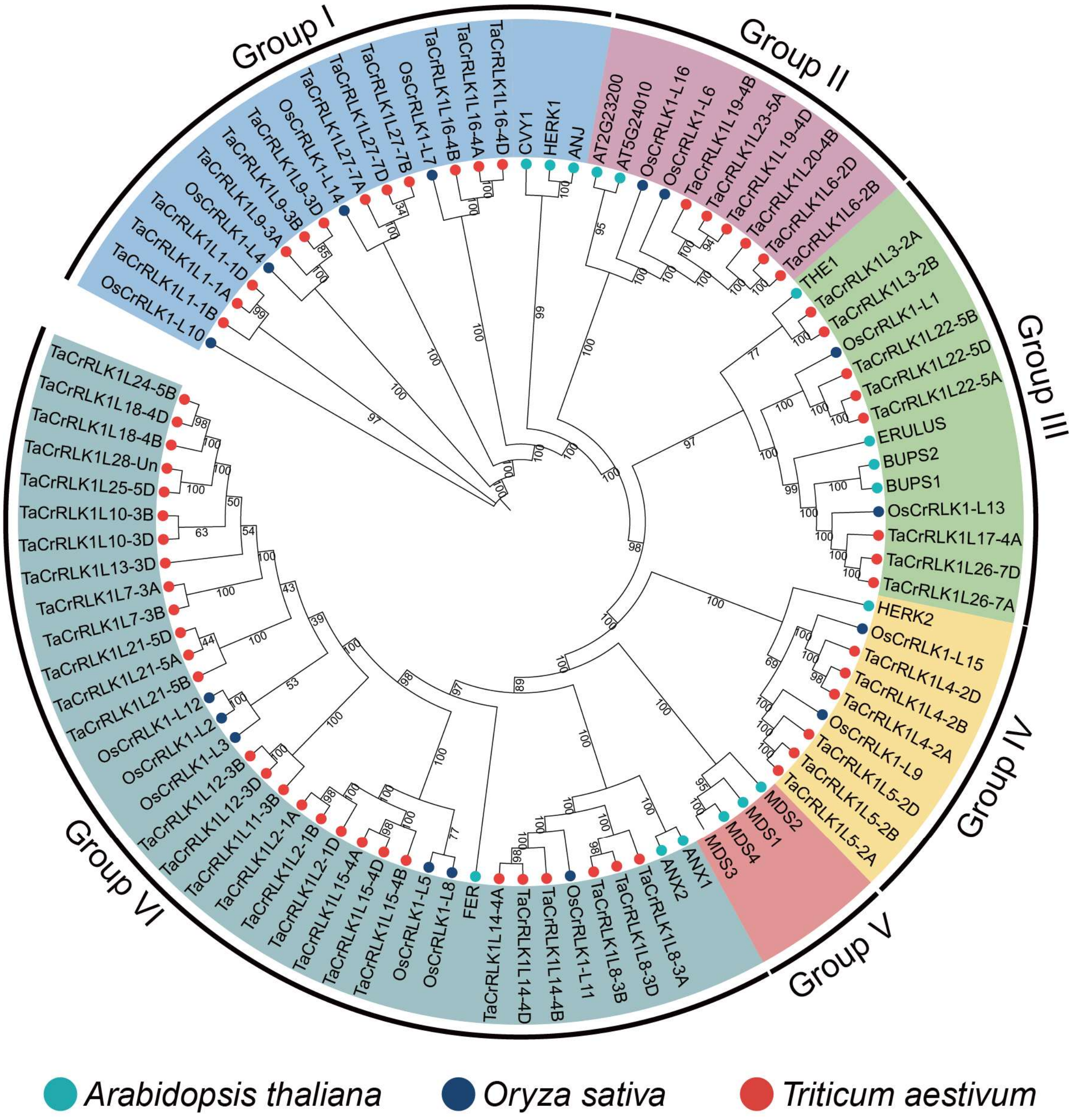

Figure S4. Phylogenetic analysis of the CrRLK1L subfamily genes in Arabidopsis, rice and wheat. The protein sequences of AtCrRLK1Ls (Cheung and Wu 2011), OsCrRLK1Ls (Nguyen et al. 2015) and TaCrRLK1Ls were aligned results using MAFFT with L-INS-I strategy. The phylogenetic tree was constructed using the IQ-TREE 2 with the maximum likelihood method based on the JTT + F + R8 substitution model and supported by 1,000 bootstrap replications.
